# Potential physiological responses contributing to the ergogenic effects of acute ischemic preconditioning during exercise: A narrative review

**DOI:** 10.3389/fphys.2022.1051529

**Published:** 2022-11-28

**Authors:** Liam O’Brien, Ira Jacobs

**Affiliations:** ^1^ Faculty of Kinesiology and Physical Education, University of Toronto, Toronto, ON, Canada; ^2^ The Tannenbaum Institute for Science in Sport, University of Toronto, Toronto, ON, Canada

**Keywords:** ischemic preconditioning, exercise, mechanism, IPC exercise, athletic performance, ergogenic aid, physiology, sport science

## Abstract

Ischemic preconditioning (IPC) has been reported to augment exercise performance, but there is considerable heterogeneity in the magnitude and frequency of performance improvements. Despite a burgeoning interest in IPC as an ergogenic aid, much is still unknown about the physiological mechanisms that mediate the observed performance enhancing effects. This narrative review collates those physiological responses to IPC reported in the IPC literature and discusses how these responses may contribute to the ergogenic effects of IPC. Specifically, this review discusses documented central and peripheral cardiovascular responses, as well as selected metabolic, neurological, and perceptual effects of IPC that have been reported in the literature.

## Introduction

Ischemic preconditioning (IPC) is a potential ergogenic technique that involves administering brief repeated episodes of non-lethal ischemia and reperfusion to the limb(s) prior to engaging in exercise. IPC is performed by temporarily occluding the flow of blood through the limb(s) *via* pressurized tourniquets for a specific duration (most commonly 5 min) followed by a period of reperfusion. This tourniquet-induced ischemia and reperfusion is then repeated for a prespecified number of intervals (most commonly 3–4 times) before commencing exercise (O’Brien and Jacobs, 2021). Although IPC is similar to the increasingly popular modality of blood flow restricted (BFR) exercise (Patterson et al., 2019)—which typically refers to low intensity aerobic or resistance exercise while inflated cuffs are applied to the exercising limb(s) to restrict blood flow during exercise–IPC differs in its application as it is employed passively prior to exercise and is assumed to require complete occlusion of the arteries supplying blood to the limb(s) for prespecified durations and intervals (O’Brien and Jacobs, 2021).

Previously, IPC has been demonstrated to have protective properties against the injurious effects of ischemia-reperfusion injury in mammalian animal models (Pérez-Pinzón, 2004; Konstantinov et al., 2005; Kristiansen et al., 2005; Wang et al., 2016) and has been shown in humans to improve various clinical outcomes such as protection against post-ischemia-reperfusion endothelial dysfunction, and post-surgical acute kidney injury, among others (Luca et al., 2013; Khaliulin et al., 2019; Gu et al., 2021; Liu et al., 2021). In sport and exercise research literature, IPC has been repeatedly reported to improve athletic performance using a variety of maximal exercise intensities and modalities such as running, swimming, or cycling lasting from about 30 s to 20 min (Jean-St-Michel et al., 2011; Bailey et al., 2012b; Cruz et al., 2015, 2016; Kraus et al., 2015; Cocking et al., 2018b; Paradis-Deschênes et al., 2018). However, the consistency and magnitude of such ergogenic responses is highly variable throughout the literature and merits further attention (For review of the ergogenic responses to IPC see: Marocolo et al., 2015a; Marocolo et al., 2019; Incognito et al., 2016; Salvador et al., 2016; Caru et al., 2019).

It has previously been suggested that the training status or sex of the participant may influence the probability of IPC ergogenicity (Gibson et al., 2013; Tocco et al., 2015; Paradis-Deschênes et al., 2017), although there is conflicting evidence (Jean-St-Michel et al., 2011). The possibility has also been raised that there are IPC non-responders (Slysz and Burr, 2018; Slysz et al., 2019). Others have suggested that, due to a paucity of well-blinded investigations, IPC-mediated performance enhancement may be a placebo effect (Marocolo et al., 2015b; Marocolo et al., 2016; Sabino-Carvalho et al., 2017; de Souza et al., 2021). However, both placebo- and nocebo-controlled studies have still found ergogenic benefits of IPC which suggests that a placebo effect alone is unlikely to explain the entirety of observed ergogenic responses (Ferreira et al., 2016; Cheung et al., 2020). We recently reviewed the burgeoning literature on IPC and exercise performance and identified various methodological considerations that may further contribute to heterogenous IPC response rates including differences between or lack of controls of the specific IPC protocol, the tourniquet pressures used, the location of the IPC stimulus, and the time between IPC administration and exercise (O’Brien and Jacobs, 2021).

There is no doubt that there has been growing interest from sport and exercise researchers and practitioners in IPC as a promising ergogenic aid, and there is also no doubt that the mechanistic explanations for the observed performance enhancing effects of the treatment are incompletely understood. Thus, a comprehensive understanding of the physiology of IPC and exercise is imperative to maximizing the likelihood of a positive ergogenic response occurring. While several published reviews have summarized the observed ergogenic effects or methodological variations in IPC exercise studies, at the time of preparing this paper we could find no review that has collated the myriad physiological mediators purported to be the mechanisms that explain the observed ergogenic effects attributed to IPC. Therefore, this narrative review aims to summarize the current evidence of the effects of an acute bout of IPC on the physiological responses to exercise, regardless of whether an ergogenic effect was elicited. Specifically, this report discusses the documented cardiovascular, metabolic, neural, and perceptual responses that occur *via* IPC and how they may contribute to enhancing exercise performance outside of the laboratory. The primary objective of this review is to provide the reader with an up-to-date report on the physiology of IPC-exercise that will help to inform future IPC practitioners when using IPC for the purposes of performance enhancement during exercise.

## Potential mechanisms for IPC-induced ergogenicity

**FIGURE 1:**
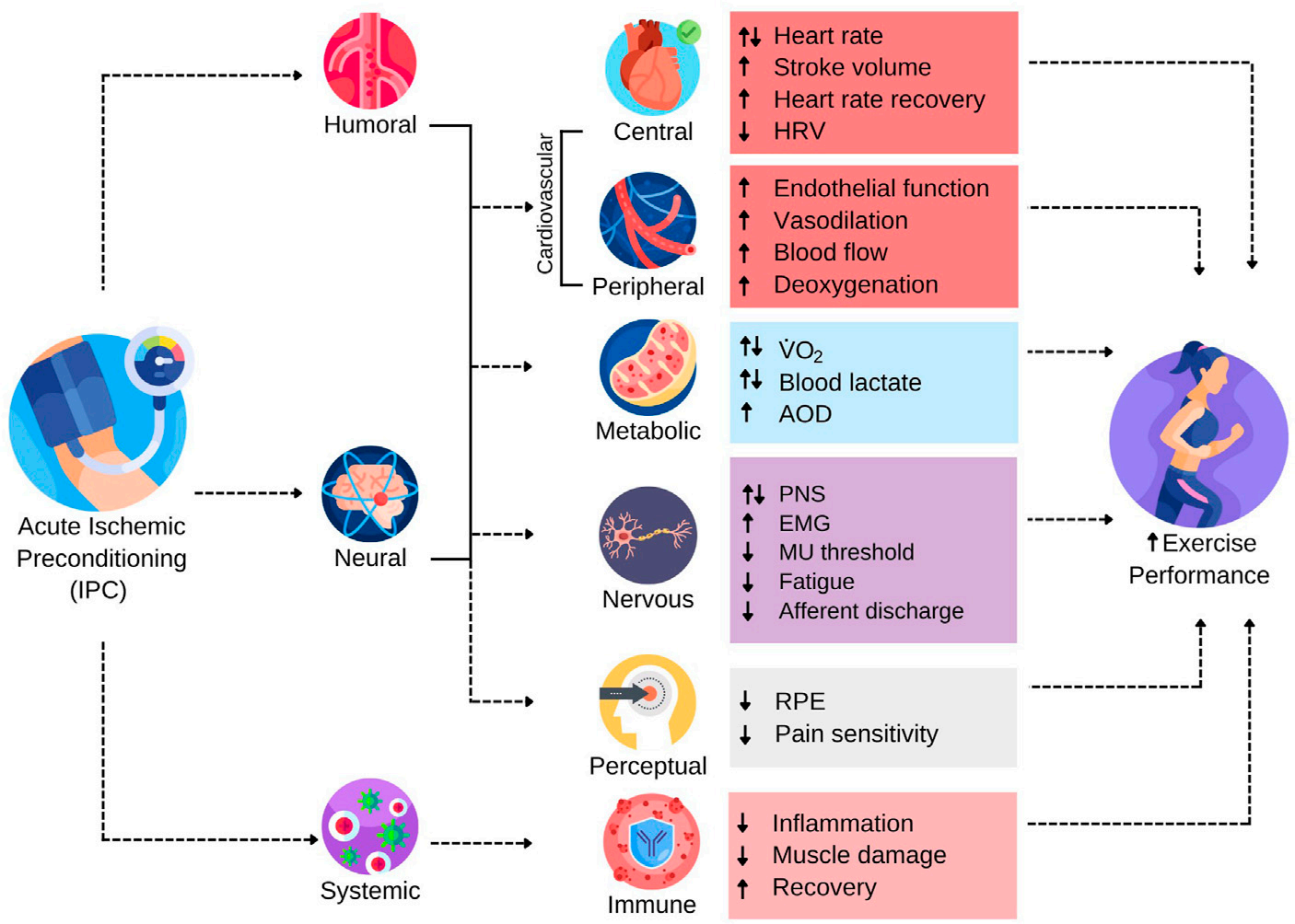
The Range of Reported Potential Triggers and Subsequent Responses Contributing to Ergogenic Effects of IPC. AOD, accumulated oxygen deficit; EMG, electromyography; HRV, heart rate variability; MU, motor unit; PNS, peripheral nervous system; RPE, rating of perceived exertion; $\dot{V}O_{2}$ , oxygen consumption. Arrows indicate directionality of documented changes in the literature (See Supplementary Tables and body of text for further information on the strength of evidence for each of these responses).

Figure 1 has been constructed to provide a schematic depiction of many of the triggers and subsequent physiological responses raised in the literature that have been purported as contributing to IPC-induced ergogenicity. The reader should be aware that each of the relationships shown in Figure 1 are accompanied by varying degrees of evidence, which is a main focus of this manuscript.

Much of the foundational work on underlying IPC mechanisms arose from clinical and animal models that investigated the role of remote IPC (RIPC) in preventing or remediating ischemia-reperfusion injury in various tissue types (Murry et al., 1986; Dickson et al., 1999, 2000; Kristiansen et al., 2005). The protective effects of IPC are reputed to involve humoral, neural, and systemic triggers that initiate a cascade of physiological responses that render tissues more tolerant to subsequent ischemia (Hausenloy and Yellon, 2008). The downstream biochemical pathways purportedly responsible for the protective effects of IPC are complex and have been reviewed previously (Heusch et al., 2015; Kleinbongard et al., 2017; Lang et al., 2022). Presently, it is not known how or if these protective biochemical pathways interact with exercise to promote performance enhancement, or whether the ergogenic effects involve distinctly different physiological responses. Nevertheless, humoral, neural, and systemic responses are putative triggers to the ergogenic effects of IPC (Figure 1). Autacoids released from the ischemic tissue appear to stimulate cardiovascular and neural responses that may affect the physiological response to exercise (Amann et al., 2011; Bouffard et al., 2021). Moreover, IPC has been shown to have systemic effects that modulate the inflammatory response to exercise and attenuate muscle damage, which may indicate the utility of IPC for facilitating training adaptations by reducing cellular damage and augmenting recovery potential from an exercise bout (Arriel et al., 2018; Mieszkowski et al., 2020; Patterson et al., 2021).

## Effects of IPC on central and peripheral cardiovascular responses

### Central cardiovascular responses

It is important to consider if the well-documented cardioprotective effects of RIPC (Serejo et al., 2007; Rassaf et al., 2014; Singh et al., 2017) mean that RIPC also has beneficial cardiovascular effects on exercise performance. Supplementary Table S1 summarizes the central and peripheral cardiovascular and hemodynamic responses documented following an acute bout of IPC. Changes in central cardiovascular responses following IPC have been observed previously but are not a typical response. For example, in two studies, resting heart rate (HR) was shown to decrease following IPC (Enko et al., 2011; Morley et al., 2021). At the same submaximal exercise intensity, IPC was reported in one study to be associated with a decreased HR (Arriel et al., 2020), whereas another study found an increase in exercising HR at 30% of maximal oxygen consumption ( $\dot{V}O_{2\max}$ ) (Clevidence et al., 2012). During maximal intensity exercise, IPC resulted in a lower HR despite no performance differences during judo-specific fitness testing (Ceylan and Franchini, 2022) and increased maximal HR along with exercise endurance and work output during incremental cycling exercise (Crisafulli et al., 2011). Another study found IPC resulted in an increased HR at various time points during 5 km cycling time trials at low altitude with no change in performance, whereas at moderate altitude performance was increased with decreases in HR and a concomitant increase in stroke volume from IPC (Paradis-Deschênes et al., 2018). While such responses have been observed after IPC, most investigations reported that IPC had no effect on cardiac output, HR, or stroke volume, regardless of exercise intensity, modality, or performance enhancement (see Supplementary Table S1 for references).

Similarly, when examining the effects on blood pressure responses, a minority of studies listed in Supplementary Table S1 reported an effect of IPC. Foster et al. (2011) reported that lower limb IPC attenuated the typical increase in systolic blood pressure during hypoxic exercise. Using a protocol where single arm IPC occurred before metaboreflex activation elicited *via* 3 min of post exercise muscle ischemia, Mulliri et al. (2016) found that the mean arterial pressure response was reduced compared to post-exercise muscle ischemia without preceding IPC. During isometric plantar flexion exercise to exhaustion, Pereira et al. (2020) reported a sex-specific response of IPC, with increased mean arterial pressure responses occurring only in male participants. One investigation reported reduced post-exercise blood pressure following resistance exercise preceded by IPC compared to a control condition (Panza et al., 2020), which may constitute safety concerns for using IPC in populations prone to post-exercise syncope. Furthermore, Barbosa et al. (2015) found increased mean blood pressure at peak exercise intensity during handgrip exercise preceded by IPC, which contrasts with reports finding no changes in blood pressure during static or dynamic handgrip exercise (Horiuchi et al., 2015; Incognito et al., 2017).

Collectively, even with the sound theoretical basis for speculating that IPC before exercise should influence central cardiovascular responses during exercise, there is very limited supporting empirical evidence that such is the case. Thus, it is likely that physiological mechanisms other than central cardiovascular effects of IPC are primarily responsible for the ergogenic effects of IPC, while central cardiovascular responses may contribute modestly in some cases.

### Peripheral cardiovascular responses

Compared to central cardiovascular responses more robust evidence exists to postulate that peripheral cardiovascular responses contribute to IPC-induced performance enhancement. IPC has been shown to acutely affect peripheral vascular responses such as enhanced conduit artery vasodilation, endothelial function, convective O_2_ delivery, and deoxygenation kinetics at the muscle capillary interface (Loukogeorgakis et al., 2005; Enko et al., 2011; Paradis-Deschênes et al., 2016; Cocking et al., 2018a). The reader is directed to the following references for associated details of each of these phenomena (Pagliaro et al., 2003; Luca et al., 2013; Paradis-Deschênes et al., 2016, 2017; Cocking et al., 2018a; Gu et al., 2021).

Changes in blood vessel function appear to involve both local and neural mechanisms depending on the location of the IPC stimulus relative to the tissue of interest (i.e., RIPC *versus* local IPC). External compression of the peripheral blood vessels in the limb *via* IPC may augment local feedforward and feedback responses at the site of ischemia that may persist for some time following the cessation of the treatment (Tschakovsky and Pyke, 2010; Joyner and Casey, 2015; Roseguini and Laughlin, 2018). During ischemia, local vasodilators such as adenosine, bradykinin, or nitric oxide (NO) are phosphorylated in the ischemic tissue and released into systemic circulation where they promote vasodilatory responses and upregulate parasympathetic activity (Gho et al., 1996; Liem et al., 2002; Kimura et al., 2007; Enko et al., 2011). During reperfusion, the sudden and rapid restoration of blood flow to the limb may be expected to increase shear stress in the conduit arteries, promoting flow mediated dilation (FMD) in the target limb(s) (Bailey et al., 2012a). This may further stimulate NO synthase or adenosine mediated actions at the ischemic site that further contribute to changes in local vessel diameter (Rosenberry and Nelson, 2020). Together, when IPC is applied to the exercising limb(s), upstream vasoactive responses such as FMD may theoretically contribute to enhancing vascular conductance and promote greater downstream convective O_2_ delivery to the site of the active skeletal muscle, thereby enriching the pool of available O_2_ for extraction and utilization during exercise. This assumes that there is a proportional metabolic demand for O_2_ during exercise preceded by IPC, which may not be the case if ischemic protection is involved in altering metabolic efficiency (Pang et al., 1995; Addison et al., 2003; Andreas et al., 2011).

Indeed, evidence supports the above theory of local vasodilatory responses to IPC. A recent systematic review and meta-analysis by Gu et al. (2021) investigated the role of arm IPC on resting vascular endothelial function and the relationship between IPC, FMD, and brachial artery diameter. That review concluded that there are beneficial effects of IPC on endothelial function of the blood vessels by enhancing FMD. However, despite improved endothelial function and FMD, there was no effect found of IPC on brachial artery diameter. Although conducted in non-exercise studies, these findings lend support to the hypothesis of acute improvements in vascular endothelial function and our speculation that such improvements have the potential to contribute to enhanced exercise capacity. Further, one exercise study did find enlarged brachial artery diameter during rhythmic handgrip exercise preceded by IPC to the arms, which may suggest an effect of IPC on conduit artery diameter when IPC is combined with exercise (Cocking et al., 2018a). Several other investigations have demonstrated positive effects of IPC techniques on endothelial function but more research is necessary to elucidate the role of IPC in modulating endothelial responses during exercise (Loukogeorgakis et al., 2005; Enko et al., 2011; Manchurov et al., 2014; el Dabagh et al., 2017).

Vasoactive responses have also been observed in blood vessels surrounding tissues located remotely to the IPC stimulus; such observations cannot be plausibly be explained by local feedforward or feedback responses (Tschakovsky and Pyke, 2010; Crecelius et al., 2012). However, these observations may be indirect evidence suggestive of the existence of a neural component to altered peripheral vascular responses following RIPC, although the precise mechanisms for this response are equivocal. Demonstrating this phenomenon, Manchurov et al. (2014) reported augmented brachial artery FMD after acute RIPC to the contralateral limb with the effects persisting up to 7 d after exposure. Another study reported a larger brachial artery diameter after RIPC was applied to the contralateral limb and this response was associated with increased parasympathetic activity (Enko et al., 2011). This finding is consistent with other reports highlighting the ability of IPC techniques to blunt sympathetic nervous system activity which will be discussed later in this review (Horiuchi et al., 2015). RIPC has also been reported to sustain FMD in the contralateral limb for at least 48 h after ischemia-reperfusion injury, and these effects are purportedly neuronally mediated (Loukogeorgakis et al., 2005). Similar remote vasoactive effects have been observed in tissues more distal to the RIPC stimulus as well. For example, Bailey et al. (2012a) found that IPC applied to the lower extremities prior to 5 km running time-trials prevented the typically observed post-exercise reductions in brachial artery endothelial function during FMD tests. Another report demonstrated increased FMD in young people as well as healthy or hypertensive elderly patients following both local and remote IPC, which lends further support to local and neuronally mediated vasoactive response to IPC and RIPC (Moro et al., 2011).

The above responses provide a theoretical basis for speculating that local and neurally mediated vasoactive responses to IPC could lead to improved exercise performance. Moreover, studies assessing blood volume and deoxygenation kinetics during exercise provide additional support for this theory by demonstrating the expected downstream effects of these responses at the exercising muscle. For example, acute local IPC is often accompanied by near infrared spectroscopy-measured (NIRS) marked increases in total haemoglobin [tHb] which is an indicator of total blood volume (Paradis-Deschênes et al., 2016, 2017), and deoxygenated haemoglobin [HHb] which is an indicator of muscle oxygen extraction (Paradis-Deschênes et al., 2016, 2017, 2020; Halley et al., 2018) in active muscle during intense exercise, although this is not always the case (Supplementary Table S1). The tissue saturation index (TSI)—which represents the equilibrium between O_2_ supply and consumption during exercise–has also been shown to be reduced along with concomitant reductions in oxygenated haemoglobin [O_2_Hb], further highlighting this deoxygenation response (Turnes et al., 2018). It is not clear for how long after the treatment protocol that enhanced muscle perfusion and deoxygenation kinetics persist, but Cunniffe et al. (2017) reported increased change in oxygenated haemoglobin [ΔO_2_Hb] in the vastus lateralis for at least 15 min after and da Mota et al. (2019) found augmented deoxygenation responses when performing exercise 2 h after IPC, which suggests some latency in the effect. Despite these examples demonstrating evidence of enhanced blood volume and deoxygenation during exercise following IPC, other studies have reported contrasting results (Seeger et al., 2017; Griffin et al., 2018; Beek et al., 2020; Cocking et al., 2020; Mota et al., 2020; Tanaka et al., 2020). One study reported greater TSI maintenance during repeated sprint running bouts, suggesting attenuated muscle deoxygenation kinetics (Patterson et al., 2015). However, these authors did not report values for [tHb], [HHb], or [O_2_Hb], which presents challenges with determining the mediator of enhanced TSI maintenance.

The peripheral hemodynamic response may be less robust during RIPC compared to local IPC, although the limited available evidence should be acknowledged here. One study found modest changes to hemodynamic variables following RIPC (Richard and Billaut, 2018), whereas another found no effect of RIPC on hemodynamic responses during rhythmic handgrip exercise (Barbosa et al., 2015). With such limited evidence on the hemodynamic responses of RIPC in exercising muscle, it would not be appropriate to propose that an effect exists. Similarly, more research is necessary to improve the understanding of the potential neural contribution to RIPC vasodilation and whether this leads to downstream enhancements in muscle perfusion and deoxygenation responses.

Taken together, a growing body of evidence summarized in Supplementary Table S1 suggests that peripheral vascular and hemodynamic responses may contribute to the ergogenic responses observed following local and remote IPC. These responses are likely mediated by several known vasoactive mechanisms such as shear mediated dilation (Gu et al., 2021), neuronally mediated vasodilation (Manchurov et al., 2014), reactive hyperemia (Rosenberry and Nelson, 2020), and increased extraction of O_2_ at the muscle-capillary interface (Paradis-Deschênes et al., 2016). Curiously, these effects are not always observed, which may be partly attributable to methodological variations between study protocols or may infer the existence of IPC non-responders (O’Brien and Jacobs, 2021).

## Effect of IPC on metabolic responses to exercise

One of the central motivations for the initial study of IPC as an ergogenic aid was its promising potential to promote enhanced metabolic efficiency in active tissues. In theory, this could be useful during exercise at terminal intensities where the magnitude of the mismatch between O_2_ supply and demand can be reduced with improved metabolic efficiency resulting in a greater endurance capacity (Joyner and Casey, 2015). Early IPC studies provided evidence that IPC modulates metabolic activity in tissues as reflected by attenuated rates of muscle lactate accumulation, as well as adenosine triphosphate and glycogen depletion during prolonged ischemic episodes in resting animal muscle (Pang et al., 1995; Addison et al., 2003). This suggests that IPC could alter oxidative efficiency or substrate utilization in active muscle during exercise. In humans, functional MRI has been used to demonstrate that IPC positively influences resting skeletal muscle metabolism by promoting increased phosphocreatine production and O_2_ consumption (Andreas et al., 2011). However, more recent evidence suggests that altered muscle energy metabolism during exercise preceded by IPC does not appear to be as promising of a potential mediator of ergogencity as was once hypothesized (Supplementary Table S2).

In exercise interventions, the metabolic responses to IPC maneuvers have been extensively measured with varying results (Supplementary Table S2). Most commonly, exercising metabolic activity has been characterized using indirect calorimetry and blood lactate measurements, although some studies have used other methods to assess additional metabolic variables. For example, blood chemistry profiles have consistently demonstrated no effect of IPC on blood glucose, pH, bicarbonate, carbon dioxide, or O_2_ saturation values after exercise (Williams et al., 2018; Halley et al., 2020; Paradis-Deschênes et al., 2020; Chen et al., 2022). Sixty-seven percent of the studies listed in Supplementary Table S2 report no effect of IPC techniques on key indices of aerobic or anaerobic metabolism, regardless of outcomes of IPC on athletic/exercise performance. Still, those studies that have reported acute alterations in aerobic and/or anaerobic exercise metabolism indicate the topic merits further interrogation.

Acute modifications to aerobic metabolism can be observed in studies assessing respiratory variables such as oxygen consumption ( $\dot{V}O_{2}$ ). One study found IPC reduced $\dot{V}O_{2}$ during a baseline low-intensity exercise period compared to a control condition (Kido et al., 2015). IPC has been shown to increase $\dot{V}O_{2peak}$ during constant load cycling (Cruz et al., 2015), $\dot{V}O_{2\max}$ during incremental exercise testing (de Groot et al., 2010), ventilation during incremental cycling (Crisafulli et al., 2011), and excess post-exercise $O_{2}$ consumption after sprint cycling exercise (Cruz et al., 2016). In other investigations, IPC has been demonstrated to influence $\dot{V}O_{2}$ kinetics by lowering $\dot{V}O_{2}$ amplitude and $\dot{V}O_{2}$ mean response time during moderate intensity exercise (Kido et al., 2015), reducing end-exercise $\dot{V}O_{2}$ , slow component amplitude, and overall gain during heavy intensity exercise (Kilding et al., 2018), and increasing the amplitude of the slow component of $\dot{V}O_{2}$ kinetics during maximal aerobic cycling (Cruz et al., 2015). Regardless of these observed responses, the reader is reminded that these are not common observations found in the literature (Supplementary Table S2).

The effect of IPC on anaerobic metabolic activity has also been extensively examined indirectly through measuring changes in blood lactate concentrations and respiratory gases. Nine of the studies listed in Supplementary Table S2 reported altered blood lactate responses following IPC whereas 34 studies found no alterations to blood lactate concentrations. Baseline blood lactate concentrations were found to be lowered *via* IPC in two studies (Paull and van Guilder, 2019; Mota et al., 2020). Bailey et al. (2012b) reported attenuated increases in submaximal blood lactate concentrations after IPC compared to a low-pressure sham which could suggest enhanced submaximal oxidative metabolism. However, no differences in submaximal gas exchange parameters were found in this same report. Other reports found post-exercise blood lactate concentrations to be significantly higher in IPC conditions after intense cycling (Cruz et al., 2015, 2016; Patterson et al., 2015) and swimming exercise (Lisbôa et al., 2017). Conversely, Gibson et al. (2015) found significantly lower blood lactate concentrations in female, but not male, participants after repeated sprint cycling. Finally, Ceylan and Franchini (2022) reported significantly lower post-exercise blood lactate concentrations after judo-specific fitness tests preceded by IPC. The accumulated oxygen deficit (AOD)—which is used as a proxy of anaerobic capacity during exercise (Noordhof et al., 2010)—has also been demonstrated to be influenced by IPC administration (Cruz et al., 2016; Paull and van Guilder, 2019; Chen et al., 2022). Cruz et al. (2016) reported increased AOD following IPC administration during sprint cycling exercise whereas Paull and Van Guilder (2019) observed RIPC to increase AOD during supramaximal running time-to-exhaustion tests. More recently, Chen et al. (2022) found both IPC and RIPC techniques to increase AOD during supramaximal running in trained runners. In contrast, one study did not find alterations to AOD following IPC administration during simulated 1,000 m kayak ergometry races (Halley et al., 2020).

Taken together, the effects of IPC on metabolic responses to exercise are widely ranging and equivocal (see Supplementary Table S2). Although early IPC studies showed promise for IPC to potentially modulate metabolic activity during exercise, most studies do not demonstrate an acute effect of IPC on indices of aerobic or anerobic metabolic activity. However, there are a notable subset of studies that have demonstrated differential metabolic responses to exercise after IPC such as changes in $\dot{V}O_{2}$ , AOD, and blood lactate concentrations, which should not be overlooked. More work is necessary to determine why these studies show altered metabolic responses while others do not.

## Effect of IPC on neurological and perceptual responses to exercise

Seeing as there is a known neural trigger from RIPC in mediating ischemic protection (Costa et al., 2001; Weinbrenner et al., 2002; Khaliulin et al., 2019), it is tempting to speculate that the nervous system contributes to the ergogenic benefit of IPC. Yet, comparatively fewer IPC-exercise investigations have directly examined the effects of IPC techniques on modulating neurological responses during exercise (Supplementary Table S3). Growing research efforts have begun to reveal the potential beneficial effects of IPC on modulating cardiac autonomic responses to exercise. These reports have shown IPC to increase heart rate recovery, and reduce cardiac parasympathetic activity and heart rate variability responses (Lopes et al., 2018; Sabino-Carvalho et al., 2019; Morley et al., 2021; da Silvas Telles et al., 2021; Telles et al., 2022). Reduced heart rate variability can be used as an indirect marker of reduced cardiac parasympathetic nervous activity (Morely et al., 2021), which could possibly attribute to increased cardiac output during IPC exercise (Crisafulli et al., 2011). Conversely, other reports have found no effect of IPC maneuvers on heart rate variability responses which indicates that more work is necessary to elucidate the neurocardiovascular response to IPC (Lopes et al., 2018; Sabino-Carvalho et al., 2019). Furthermore, IPC has been shown to augment functional sympatholysis which may contribute to enhanced peripheral vascular function during exercise (Horiuchi et al., 2015).

Based on the available literature, most studies that measure electromyographical (EMG) activity during IPC-exercise have found no effect on neuromuscular function, regardless of whether performance enhancement was found (Supplementary Table S3). Conversely, acute IPC has been shown to enhance EMG activity and delay neuromuscular fatigue in other reports (Cruz et al., 2015, 2016; Hyngstrom et al., 2018; Pethick et al., 2021). In some investigations, increases in maximal strength following IPC have been purported to involve increased neuromuscular activity and reduced motor unit recruitment thresholds (Hyngstrom et al., 2018; Durand et al., 2019). It is not clear why some reports find altered EMG activity during exercise preceded by IPC while others do not, and the interpretation of such conflicting findings is challenging. Nevertheless, the minority of studies suggesting modulated neuromuscular responses should not be overlooked. It is possible that there exists a dose-response relationship between IPC and neuromuscular responses to exercise. Recently, Morley et al. (2021) demonstrated greater perturbations on cardiac autonomic responses when combining RIPC with concurrent low intensity cycling exercise compared to traditional RIPC. Another report found the ergogenic effects of IPC were augmented by enhancing metabolic stress by walking or electrical muscle stimulation in conjunction with IPC (Slysz and Burr, 2018). Further research efforts should aim to elucidate the required metabolic stress threshold to elicit effects of IPC on physiological responses to exercise.

One neural mechanism that has been previously hypothesized to contribute to the ergogenic effect of IPC involves the desensitization of metabolically sensitive metabo-nociceptive neurons known as group III/IV muscle afferents (Crisafulli et al., 2011; Cruz et al., 2015, 2016; Salvador et al., 2016; Incognito et al., 2017). Briefly, group III/IV muscle afferents are free nerve endings that respond to perturbations in the mechanical and metabolic states of muscle, respectively (Amann et al., 2011). Feedback mechanisms *via* group III/IV muscle afferents have been shown to initiate the exercise pressor reflex, which is an essential response governing the cardiovascular response to exercise (Rowell and O’Leary, 1990; Amann et al., 2011; Nobrega et al., 2014). The exercise pressor reflex may account for some of the central and peripheral cardiovascular responses discussed earlier in this review. Group III/IV muscle afferents contribute to various essential processes of the typical physiological response to exercise, including inducing central fatigue (Sidhu et al., 2013), inhibiting α-motor neuron activation (Gandevia, 2001; Amann et al., 2011), and modulating ascending pain signalling in response to noxious metabolic stimuli produced as biproducts of muscle contraction (O’Connor and Cook, 1999; Crisafulli et al., 2006, 2008; Kaufman, 2012). Theoretically, if IPC were to reduce stimulation of these muscle afferents, exercise performance could be prolonged through attenuating central nervous system fatigue, delaying the inhibition of α-motor neurons, ameliorating the perception of pain, and/or augmenting the neuro-cardiovascular response to intense exercise.

Few studies have sought to directly investigate the relationship between IPC on discharge from group III/IV muscle afferents. One study found an attenuated hemodynamic response to metaboreflex activation during dynamic handgrip exercise preceded by IPC (Mulliri et al., 2016), whereas another report did not show IPC to reduce hemodynamic responses or central muscle sympathetic nervous activity during activation of the metaboreflex (Incognito et al., 2017). More recently, Angius et al. (2022) corroborated the previously observed reduction in hemodynamic responses during metaboreflex activation using dynamic leg extension exercise. However, these authors also reported reduced end diastolic volumes following IPC which seems to indicate that altered hemodynamic responses during metaboreflex activation were due to blunted cardiac preload rather than discharge from group III/IV muscle afferents. To the best of our knowledge, no published studies have investigated the effect of IPC on the mechanoreflex. Thus, the current evidence on the effect of IPC on group III/IV afferent discharge is insufficient to draw a conclusion about the magnitude of the potential contribution to IPC-induced ergogenicity at this time.

Perhaps lending further support to the muscle afferent hypothesis is the evidence of IPC inducing hypoalgesic effects in humans. Indeed, previous investigations have demonstrated IPC techniques to reduce pressure pain ratings, pain under ischemia, perceived pain following eccentric exercise induced muscle damage, and time spent under pain during cold-water immersion tests (Ferreira et al., 2016; Pereira et al., 2020; Slysz and Burr, 2021; Angius et al., 2022). IPC-induced hypoalgesia may be governed by endogenous substances that have antinociceptive effects on pain pathways. Metabolic mediators including adenosine, bradykinin, opioids, and endocannabinoids–all of which are involved in pain signalling during exercise (Sgherza et al., 2002; Murase et al., 2010; Aguiar et al., 2020; Hughes and Patterson, 2020; Hughes et al., 2021)—have been purported to contribute to the protective response to RIPC (Lee et al., 1996; Tomai et al., 1999; Cohen et al., 2000; Schoemaker and van Heijningen, 2000; Dickson et al., 2002; Patel et al., 2002; Hajrasouliha et al., 2008). Furthermore, the endogenous release of some of these autacoids is thought to modulate the stimulation and signalling of group III/IV afferents (Amann et al., 2009, Amann et al., 2011; Bouffard et al., 2021).

Indeed, activation of opioid and endocannabinoid receptors in the central and peripheral sites of the pain pathway such as the spine and peripheral nerves can modulate ascending and descending pain signalling (Sgherza et al., 2002; Hughes and Patterson, 2020; Hughes et al., 2021). Specifically, plasma concentrations of an opioid neuropeptide known as beta-endorphin (BE) and the endocannabinoid endogenous ligand agonist 2-arachidonoylglycerol (2-AG) have recently been shown to be elevated following BFR exercise in humans (Hughes and Patterson, 2020; Hughes et al., 2021). Although there is a distinction between BFR and IPC, the similarities between these techniques involves overlapping mechanisms (Jessee et al., 2018; Patterson et al., 2019), which may implicate the involvement of these substances in the IPC response. Briefly, BE is thought to be released by the pituitary in response to stimulation of group III/IV afferents, thereby activating the opioid system and descending pain inhibitory pathways (Thoren et al., 1990; Hughes and Patterson, 2020). 2-AG appears to involve activation of endocannabinoid receptors on A-delta and C-delta primary afferents (Koltyn et al., 2014), which, to the best of our knowledge have not been explored in IPC research. Future research efforts should aim to elucidate the potential role between the endogenous opioid and endocannabinoid systems in modulating perceived pain or discomfort during exercise, and whether this contributes to the ergogenic response.

Pain perception serves an important function in protecting the body from deleterious stimuli through promoting avoidance behaviours (Philips and Jahanshahi, 1986). Similarly, during intense exercise, perceptual markers such as rating of perceived exertion (RPE) are known to initiate exercise intolerance and similar avoidance behaviours (Noble et al., 1983; Tucker, 2009). Thus, when considering the potential hypoalgesic properties of IPC, it is tempting to speculate that IPC may also attenuate perceptual markers of exertional discomfort during exercise. If IPC indeed attenuates RPE, it may be hypothesized that exercise capacity could be enhanced by this effect. That is, by attenuating RPE at a specific absolute exercise intensity, the associated relative exercise intensity is also reduced which may be significant for prolonging endurance through elevating the threshold at which exercise intolerance occurs (Tucker, 2009). Despite similar avoidance behaviours, pain and RPE are distinct subjective perceptions (Marcora, 2009). Moreover, reduced subjective pain ratings following IPC were not shown to influence RPE during fatiguing plantar flexion exercise and did not correlate with the ergogenic effects during exercise (Pereira et al., 2020; Slysz and Burr, 2021). This suggests that altered pain sensitivity does not govern the ergogenic response which is consistent with previous assertions that afferent feedback is independent from effort perception (Marcora, 2009). Further supporting the divergence between afferent feedback and perceived effort, the available evidence suggests that IPC has menial effects on RPE. Five of the studies found in Supplementary Table S4 showed attenuated RPE during exercise preceded by IPC (Bailey et al., 2012b; Cruz et al., 2015; Paradis-Deschênes et al., 2018; Beek et al., 2020; Behrens et al., 2020), whereas 25 studies found no influence of IPC on RPE during various exercise interventions. Some of this disagreement may be due to variability in exercise protocols across interventions. For example, RPE may be more likely to be attenuated during endurance efforts where an individual is exercising near maximal aerobic capacity *versus* supramaximal intensities where perceived effort should already be maximized. Nevertheless, most research efforts show no effect of IPC on RPE.

The effects of IPC on neural and perceptual responses to exercise are still being probed. Currently, there is no consistently reported effect of IPC on neural responses, however, a notable body of evidence suggests some relationship. Moreover, the majority of studies that have examined IPC and RPE have found no evidence to postulate that IPC modulates the perception of discomfort during exercise. Future research efforts should aim to generate more data on the neural effects of IPC to better characterize the potential contribution to IPC ergogenicity.

## Conclusion

The present review aimed to summarize the myriad physiological responses measured during acute IPC exercise interventions and to contemplate how such responses may contribute to enhancing exercise performance. Acute bouts of IPC have been demonstrated to modulate cardiovascular, hemodynamic, metabolic, neurological, and perceptual responses during subsequent acute exercise. However, the reproducibility and synchroneity among these responses to IPC are equivocal for reasons that remain unclear. O’Brien and Jacobs (2021) previously reviewed the widely varying methodologies extant in the IPC exercise literature, and discussed how that methodological heterogeneity likely explains some the equivocal nature of the related research findings. Thus the present review does not address the methodological differences in IPC among the studies included in the review, but instead focused on reviewing the purported physiological mediators of the effects of IPC on exercise performance.

Based on the widely varying mechanisms reported in this review as primarily responsible for mediating IPC-induced ergogenic effects, it is tempting to speculate that an “entourage” effect, i.e., a combined effect of several mediators is the explanation for observed performance enhancements. Future efforts should aim to assess the relative importance and contributions of the identified physiological and biochemical mediators as well as the necessary methodological strategies for optimizing the conditions for the most important mediators to maximize the efficacy of the treatment on performance outcomes.
