# Supplementary material for: Potential physiological responses contributing to the ergogenic effects of acute ischemic preconditioning during exercise: A narrative review
Source: Front Physiol. 2022 Nov 28;13:1051529. doi: 10.3389/fphys.2022.1051529 (PMC9742576; doi:10.3389/fphys.2022.1051529)
Supplement: Supplementary file 1 [file Table1.DOCX]

| **Supplementary Table 1 - Cardiovascular and Hemodynamic Responses to Acute Remote or Local IPC** | | | | | | |
| --- | --- | --- | --- | --- | --- | --- |
| **Study** | **Subjects** | **Exercise** | **Type of IPC** | **Protocol** | **Effect on Performance (+/-/~)** | **Findings** |
| Angius et al. (2022) | M13; F4, healthy volunteers | 3 min KE at 70% *W*max + PEMI | IPC | 3 x 5 min at 220 mmHg (exercising thigh) | NA | During metaboreflex activation via PEMI, SV, $\dot{\text{Q}}$, SV/LVET, and EDV were all lower relative to pre-PEMI in the IPC group. No changes in these variables were observed in the SHAM condition. There was no effect of IPC and PEMI on HR, or SVR compared to SHAM. During exercise, there were no effects of IPC on HR, SV, $\dot{\text{Q}}$, EDV, LVET, SV/LVET, SVR, SAP, DAP, or MAP compared to SHAM. |
| Arriel et al. (2018) | M28, trained cyclists | Incremental exercise test (Cycling) | IPC | Post Exercise:  2 x 5 min at 50 mmHg > SBP (alternate thighs) 5 x 2 min at 50 mmHg > SBP (alternate thighs) | + | IPC applied after exercise maintained performance on incremental exercise test 24 hrs post-exercise compared to SHAM. HR_max_ and HR_mean_ were not different between baseline and 24 h post in the IPC 2 x 5 and IPC 5 x 2 conditions, but HR_max_ was lower 24 h post in the SHAM condition, likely due to worsened performance. |
| Arriel et al. (2020) | 18 recreationally trained cyclists (gender not specified) | Incremental exercise test (Cycling) | IPC | 2 x 5 min at 50 mmHg > SBP (alternate thighs) | + | IPC decreased HR at submaximal point during the incremental cycling test. IPC promoted faster HR recovery during the first minute of exercise recovery compared to baseline |
| Bailey et al. (2012a) | M13, healthy volunteers | Graded exercise test + 5 km TT (Running) | IPC | 4 x 4 min at 220 mmHg (thighs) | + | IPC had no effect on HRmax during graded exercise test or 5 km running TTs. RIPC of the lower limb maintained post-exercise brachial artery FMD to pre-exercise levels compared to the SHAM condition which saw a reduction post-exercise. |
| Bailey et al. (2012b) | M13, healthy volunteers | Incremental exercise test + 5 km TT (Running) | IPC | 4 x 5 min at 220 mmHg | + | There was no effect of IPC on HR at various running speeds or checkpoints during 5 km TTs compared to the control condition. |
| Barbosa et al. (2015) | M9; M13, physically active volunteers | Rhythmic handgrip TTE at 45% MVC | RIPC | 3 x 5 min at 200 mmHg (thighs) | + | During fatiguing handgrip exercise, RIPC did not affect brachial artery diameter, blood velocity, blood flow, vascular conductance, or ΔHHb compared to CON. During exercise, the increases in HR and MAP were not different between RIPC and CON conditions. At peak exercise, HR was the same between RIPC and CON, but MAP was higher in RIPC compared to CON. |
| Behrens et al. (2020) | M16, recreationally active volunteers | Isometric KE TTE at 20% MVC | IPC | 3 x 5 min at LOP (thighs) | ~ | During fatiguing isometric contractions, there were no differences in ΔSmO_2_, or ΔtHb of the vastus lateralis between IPC and SHAM conditions. Similarly, there were no differences in ΔSmO_2_ or ΔtHb in IPC responders in this study. |
| Caru et al. (2016) | M9; F8, amateur triathletes | Steady state test at 75% and 115% GET (Cycling) | RIPC | 4 x 5 min at 50 mmHg > SBP (right arm) | NA | RIPC significantly shortened QT intervals during exercise compared to CON as well as during recovery which was not present at rest. |
| Ceylan and Franchini, (2022) | M10, elite judo athletes | Special judo fitness test | IPC | 3 x 5 min at 220 mmHg (alternate thighs) | ~ | HR was lower in the IPC condition compared to the CON condition during the judo specific test. SBP and DBP were not different between conditions during the judo specific test. |
| Clevidence et al. (2012) | M12, amateur cyclists | Incremental exercise test (Cycling) | IPC | 3 x 5 min at 220 mmHg (alternate thighs) | ~ | During cycling exercise, HR at 30% of $\dot{\text{V}}$O2max was significantly higher in IPC compared to CON condition. HR was not different between conditions at 50%, 70%, or 90% V $\dot{\text{V}}$O2max. |
| Cocking et al. (2017) | 14 recreationally to well-trained cyclists (gender not specified) | 1 h TT (Cycling) | IPC & RIPC | 4 x 5 min at 220 mmHg (alternate thighs/arms) | ~ | There was no effect of RIPC on SBP, DBP, or mean HR during 1 hr cycling TTs. Post-exercise cardiac troponin levels were lower following RIPC compared to SHAM. There was no effect of RIPC on NT-proBNP, left ventricular EF, LVEDV, RVFAC, E/é ratio, E’ in the LV, peak Ɛ, early diastolic strain rate, or SSR. LV longitudinal ASR was lower in the RIPC condition. |
| Cocking et al. (2018a) | M18, recreationally active volunteers | 30 min submaximal unilateral handgrip exercise at 25% MVC | IPC + RIPC | 4 x 5 min at 220 mmHg (thighs)  4 x 5 min at 220 mmHg (arms) | ~ | IPC increased brachial artery diameter during exercise compared to RIPC, but blood flow was similar between conditions. There was no main effect of condition on blood flow. Post-exercise FMD was similar across conditions. |
| Cocking et al. (2018b) | 12 trained cyclists (gender not specified) | 375 kJ TT (Cycling) | IPC + RIPC | 4 x 5 min at 220 mmHg (thighs) 8 x 5 min at 220 mmHg (thighs)  4 x 5 min at 220 mmHg (thigh)  4 x 5 min at 220 mmHg (arms) | + | Despite performance improvements, none of the IPC maneuvers influenced HR at various time points of the TT. |
| Cocking et al. (2021) | M11, trained cyclists | 10 x 6 s sprints (Cycling) | IPC | 4 x 5 min at 220 mmHg (thighs) | ~ | During repeated sprint cycling performance, there was no difference between IPC and SHAM with respect to deoxygenation or reoxygenation amplitudes or slopes when assessing NIRS variables. |
| Crisafulli et al. (2011) | M17, healthy volunteers | Incremental exercise test + TTE (Cycling) | IPC | 3 x 5 min at 50 mmHg > SBP (thighs) before or after exercise | + | Both IPC and EIPC protocols increased HRmax during incremental exercise test. Neither IPC nor EIPC had an effect on SV_max_, $\dot{\text{Q}}$_max_, MAP_max_, or SVR_max_ during the incremental exercise test or the all-out test. |
| Cruz et al. (2015) | 12 recreationally trained cyclists (gender not specified) | TTE at 100% PPO (Cycling) | IPC | 4 x 5 min at 220 mmHg (thighs) | + | IPC had no effect on peak HR during maximal constant load cycling exercise. |
| da Mota et al. (2019) | M13, healthy volunteers | 2 x 5 km TT (Cycling) | IPC | 3 x 5 min at 220 mmHg (thighs | + | There were no differences in HRpeak or HRmean during the two TT between IPC and SHAM. IPC led to greater ΔTSI%, ΔO_2_Hb ΔHHb, and ΔtHb compared to SHAM. |
| de Groot et al. (2010) | M12; F3, well-trained cyclists | Incremental exercise test (Cycling) | IPC | 3 x 5 min at 220 mmHg (thighs) | + | IPC had no effect on HRmax, BPrest, or BPmax. |
| El Messaoudi et al. (2013) | M10; F10, healthy volunteers | 70 min at 85% HR_max_ + TTE at 95% HR_max_ (Cycling) | RIPC | 3 x 5 min at 200 mmHg (arms) | ~ | Following cycling TTE tests, there were no differences in circulating hs-troponin, NT-Pro-BNP, or CK levels between RIPC and CON conditions. |
| Foster et al. (2011) | M6; F2, trained cyclists | 100 kJ TT (Cycling) | IPC | 4 x 5 min at 20 mmHg > SBP (unilateral thigh) | ~ | IPC attenuated the normal hypoxic increase of pulmonary SBP. There was no effect of IPC, either in hypoxia or normoxia, on HR_rest_, O_2_ saturation, tricuspid regurgitation velocity, pulmonary SBP, estimated pulmonary vascular resistance, tricuspid annulus S wave, or $\dot{\text{Q}}$. During the time-trial, IPC had no effect on HR responses in normoxia or hypoxia. |
| Garcia et al. (2017) | M8, subelite rugby players | Agility T-Test + countermovement jump + 30 s jump test | IPC | 3 x 5 min at 220 mmHg (thighs) | ~ | IPC had no effect on HR_mean_ during rugby-specific exercise protocol |
| Griffin et al. (2018) | M12, recreationally active volunteers | 3 min sprint (Cycling) | IPC | 4 x 5 min at 220 mmHg (thighs) | + | During a 3 min cycling sprint, there were no differences in HR_max_ or HR_mean_ after receiving IPC compared to the SHAM condition. There were no differences in baseline or exercising ΔTSI values during the all-out test between conditions, and no differences for HHb TD + τ during exercise |
| Griffin et al. (2019) | M12, recreationally active volunteers | Repeat sprint (Running) | IPC + RIPC | 4 x 5 min at 220 mmHg (arms + thighs) | ~ | IPC did not affect baseline TSI. IPC-arm produced greater reductions in TSI compared to the IPC-leg condition. During exercise, there was no effect of IPC on ΔTSI during the sprints, or during recovery between sprints. |
| Halley et al. (2020) | M8, well trained kayakers | Repeated 1000m simulated kayak races (Kayaking) | IPC | 4 x 5 min at 220 mmHg (alternate thighs) 40 min before TT1 or 40 min before TT1 and 20 min before TT2 | + | During successive 1000m kayak TTs, no changes were observed in SaO_2_ between IPC1, IPC2, or CON. |
| Hittinger et al. (2014) | M15, highly trained cyclists | Incremental exercise test at sea level and simulated altitude (Cycling) | IPC | 4 x 5 min at 10 - 20 mmHg > SBP (thighs) | ~ | During incremental exercise testing at sea level and simulated altitude, IPC had no effect on HR_peak_, SV_peak_, $\dot{\text{Q}}$_peak_, or SpO_2_ compared to the CON condition. |
| Horiuchi et al. (2015) | M15, healthy volunteers | Dynamic handgrip exercise at 10% and 25% MVC | IPC | 4 x 5 min at 220 mmHg (unilateral arm) | NA | IPC augmented the decrease in O_2_Hb in response to a cold pressor test compared to CON. During hand grip exercise at 25% MVC, O_2_Hb in response to cold pressor test increased significantly more when preceded by IPC than CON. IPC did not affect HR, MAP, or skin blood flow at rest or during exercise. |
| Huang et al. (2020) | M14, healthy volunteers | Isokinetic knee extension/flexion strength and endurance | IPC | 3 x 5 min at 50 mmHg > SBP (thighs) | ~ | IPC increased resting tHb and improved exercising tissue oxygen uptake indicated by ΔSaO_2_, ΔHHb, and ΔO_2_Hb during isokinetic muscular endurance test. |
| Incognito et al. (2017) | M13, healthy volunteers | 2 min static hand grip exercise at 30% MVC + 3 min PECO | IPC | 3 x 5 min at 200 mmHg (left arm) | NA | At rest, MAP was lower following IPC compared to the SHAM condition. During the static handgrip exercise and PECO measurements, there were no differences in PRE and POST values for MAP, HR, SV, $\dot{\text{Q}}$, and TVC responses between IPC and SHAM conditions. |
| James et al. (2016) | M11, recreational club runners | Incremental exercise test at 32°C (Running) | IPC | 4 x 5 min at 220 mmHg (thighs) | ~ | At rest, IPC had no effect on HR or skin, core, thigh, calf, or muscle temperatures in hyperthermia. During exercise, HR was similar throughout the graded exercise tests between IPC and CON. |
| Jean-St-Michel et al. (2011) | M8; F8, national and internationally competitive swimmers | 7 x 200 m submaximal + 100/200 m TT (Swimming) | RIPC | 4 x 5 min at 15 mmHg > SBP (arms) | + | IPC did not affect HR during 100m and 200m swimming TTs. IPC blood from athletic and non-athletic controls significantly reduced mouse myocardial infarct area compared to non-IPC condition. |
| Kaur et al. (2017) | M12; F6, recreationally active runners | Incremental submaximal (Running) | IPC | 3 x 5 min at 220 mmHg (thighs) | ~ | IPC had no effect on BP_rest_ or HR_rest_. During steady state exercise, there was no difference in HR at various submaximal intensities between IPC and SHAM. |
| Kido et al. (2015) | M15, recreationally active volunteers | Work-to-work test (Cycling) | IPC | 3 x 5 min at > 300 mmHg (thighs) | + | There was no effect of IPC on HR_rest_ or exercising HR compared to CON. ΔHHb was significantly faster in IPC compared to CON at moderate- and severe-intensity exercise. |
| Kjeld et al. (2014) | M20; F5, competitive diving and rowing athletes | Static/dynamic apnea + 1000 m TT (Rowing) | RIPC | 4 x 5 min at 40 mmHg > SBP (nondominant arm) | + | IPC reduced forearm SmO_2_ during a breath hold compared to CON and prolonged breath hold duration. |
| Libonati et al. (1998) | M6; F5, healthy volunteers | 15 x isometric wrist flexion MVC | IPC | 1 x 2 min at 200 mmHg (arm) | + | Following IPC administration, there was a 3-to-4-fold increase in hyperemic blood flow. |
| Lopes et al. (2018) | M15, recreationally active volunteers | Repeated shuttle sprint (Running) | IPC | 3 x 5 min at 220 mmHg (alternate thighs) | ~ | IPC did not change HRR within the first 30 s of recovery, but increased HRR60s after all sets of sprints. Long term HRR and time-domain heart rate variability were not changed during the recovery. Exercising HR_peak_ was also unaffected by IPC. |
| Marshall et al. (2020) | M18, recreationally active volunteers | 5 x 5 s repeat sprints at 150% *W*max (Cycling | IPC | 3 x 5 min at 220 mmHg (alternate thighs) | ~ | During repeated sprints, no difference was observed between conditions in muscle TSI. There was a set × condition interaction found in ΔHHb with values being enhanced by IPC during sets 3 – 5 compared to the SHAM. |
| Martin et al. (2021) | M14; F1, Chronic heart failure patients | Six-minute walk test (walking) | RIPC | 4 x 5 min at 20 mmHg > SBP (right arm) | ~ | HR, and SmO_2_ were not different between RIPC and SHAM during the 6MWT |
| McIlvenna et al. (2019) | M10, Competitive cyclists | Incremental exercise test + 16.1 km TT (Cycling) | IPC | 4 x 5 min at 180 mmHg (thighs) | ~ | There was no effect of IPC+nitrate or IPC+placebo supplementation on ΔHHb, ΔO_2_Hb, or ΔTSI during cycling TT compared to baseline. |
| Morley et al. (2021) | M8; F5, recreationally active volunteers | 4 x 5 min constant load cycling + orthostatic challenge | IPC | 4 x 5 min at LOP (thighs) | NA | The normal RIPC condition reduced resting HR and supressed HR 30 s following orthostatic challenge in comparison to the CON condition. RIPC_aug_ elevated HR compared to both CON and RIPC prior to and during the orthostatic challenge. LnSDNN and LnHfa power were reduced by RIPCaug on the morning of the treatment compared to RIPC and CON. |
| Mota et al. (2020) | F20, healthy volunteers | 3 min sprint (Arm Cycling) | IPC | 3 x 3 min (2 min off) at 50 mmHg > SBP (arms) | ~ | There was no effect of IPC treatment on HR_mean_ or HR_peak_ during 3 min arm cycling sprint. IPC had no effect on exercising ΔHHb or ΔO_2_Hb compared to SHAM. |
| Mulliri et al. (2016) | M14, healthy volunteers | Dynamic handgrip exercise at 45% MVC TTE | IPC | 3 x 5 min at 50 mmHg > SBP (arm) | NA | HR, SV, and $\dot{\text{Q}}$ were not affected by IPC-PEMI at rest, during exercise, or during the post-exercise recovery period when compared to CON and PEMI. The PEMI test induced a higher SV response compared with the IPC-PEMI test. After IPC-PEMI, the MAP response was reduced compared with the PEMI test. This was due to an impaired venous return that impaired SV during the IPC-PEMI more than during the PEMI test alone. |
| Panza et al. (2020) | M16, resistance trained volunteers | Multi-joint resistance exercise at 80% 1 RM to failure | RIPC | 4 x 5 min at 220 mmHg (alternate arms) | NA | Compared to the SHAM+RE and RE conditions, IPC applied before resistance exercise significantly reduced SBP, DBP, and MBP. IPC+RE potentiated a greater magnitude and duration of post-exercise hypotension compared to SHAM+RE and RE. |
| Paradis-Deschênes et al. (2018) | M13, trained cyclists | 5 km TT at low + moderate + high altitude (Cycling) | IPC | 3 x 5 min at 220 mmHg (alternate thighs) | + | During 5 km cycling TTs, IPC possibly improved $\dot{\text{Q}}$ at low altitude but did not alter SpO_2_, muscle or muscle TSI. IPC likely enhanced SpO_2_ and decreased TSI at moderate altitude. At low altitude, IPC increased HR whereas it decreased HR at moderate altitude with an increase in SV |
| Paradis-Deschênes et al. (2020) | M9, trained cyclists, runners, and triathletes | Repeat 5 km TT (Cycling) | IPC | 3 x 5 min at 220 mmHg (alternate thighs) between 5 km TTs | + | During repeat 5 km cycling TTs, there were no differences in SpO_2_, HR, ΔtHb, ΔO_2_Hb, and ΔHHb between IPC, active recovery, or neuromuscular electrical stimulation techniques. |
| Paradis-Deschênes et al. (2016) | M10, strength trained volunteers | 5 x 5 MVC KE | IPC | 3 x 5 min at 200 mmHg (right thigh) | + | IPC likely increased muscle tHb at rest. During exercise, IPC increased ΔHHb compared to SHAM. Between sets, IPC increased tHb compared to SHAM. |
| Paradis-Deschênes et al. (2017) | M9; F8, strength trained volunteers | 5 x 5 MVC KE | IPC | 3 x 5 min at 200 mmHg (right thigh) | + | IPC induced different changes between sexes for average muscle O_2_ uptake during maximal contractions compared to SHAM. Males increased ΔHHb whereas females decreased ΔHHb. IPC increased muscle tHb at rest and during recovery in both sexes. |
| Patterson et al. (2015) | M14, recreationally active | 12 x 6 s repeat sprinting (Cycling) | IPC | 4 x 5 min at 220 mmHg (thighs) | + | Resting TSI was not different between IPC and PLA. During exercise, IPC provided a greater maintenance of TSI compared to PLA. |
| Pereira et al. (2020) | M10; F10, healthy volunteers | Isometric plantarflexion TTE at 20% MVC | IPC & RIPC | 3 x 5 min at 225 mmHg (non-dominant thigh/upper arm) | + | IPC exposed sex differences in arterial pressure during fatiguing isometric plantarflexion contractions, evidenced by greater MAP in males compared to females. |
| Pethick et al. (2021) | M6; F4, healthy volunteers | Intermittent isometric knee extension contractions to failure at 40% MVC | IPC | 3 x 5 min at 224 mmHg (unilateral thigh) | + | In response to fatiguing KE exercise, the rate of increase in m$\dot{\text{V}}$O_2_ was significantly attenuated in the IPC condition compared to the SHAM condition. |
| Richard and Billaut, (2018) | M7; F2, elite long-track speed skaters | 1000 m TT (Speed Skating) | RIPC | 3 x 5 min at 30 mmHg > SBP (alternate arm) | ~ | RIPC had negligible effects on NIRS variables. In a subgroup of sprinters, RIPC likely lowered TSI at the beginning of the TT and likely increased HHb at the beginning, middle, and end of the trial. In the middle section of the trial, these changes were concomitant with a possible increase in tHb. |
| Sabino-Carvalho et al. (2017) | M14; F4, competitive middle- long-distance runners | Discontinuous incremental exercise (Running) | IPC | 4 x 5 min at 220 mmHg (alternate thighs) | ~ | HR_max_ during incremental test and supramaximal test were not different between IPC, SHAM, or CON. |
| Sabino-Carvalho et al. (2019) | M11; F4, competitive middle- long-distance runners | Discontinuous incremental exercise (Running) | IPC | 4 x 5 min at 220 mmHg (alternate thighs) | NA | Respiratory sinus arrhythmia and HRV were similar among IPC, SHAM, and CON interventions at pre- and post-intervention assessments. T30 was similar among interventions but IPC increased HRR30s at 70% and 75% of maximal effort vs SHAM and CT. IPC did not change resting cardiac vagal control, but did boost fast post-exercise cardiac vagal reactivation at exercise intensities below lactate threshold in endurance runners. |
| Seeger et al. (2017) | M10; F2, healthy volunteers | 5 km TT (Running) | IPC | 4 x 5 min at 220 mmHg (thighs) before or 24 h before exercise | ~ | There was no difference in exercising HR or TSI between IPC, 24-IPC, and CON conditions. |
| Seeley and Jacobs, (2021) | 12 recreationally trained volunteers (gender not specified) | 5 x 60 s sprints at 100% W_peak_ w/ 120 s active recovery at 30% W_peak_ (Cycling) | IPC | 4 x 5 min at 220 mmHg (alternating thigh) 5 min and 45 min before exercise | NA | IPC45 significantly enhanced TSI during active recovery between sprinting intervals compared to a 5 min delay and an identical SHAM. Exercising $\dot{\text{Q}}$, HR, and SV were not different between conditions. |
| Tanaka et al. (2016) | M12, healthy volunteers | Isometric KE TTE at 20% MVC | IPC | 3 x 5 min at > 300 mmHg (right thigh) | + | ΔHHb of the quadriceps during fatiguing isometric knee extension exercise was significantly faster following IPC. |
| Tanaka et al. (2020) | M14, healthy volunteers | Isometric KE TTE at 20% MVC | RIPC | 3 x 5 min at 300 mmHg (left/non-exercising thigh) | ~ | ΔHHb of the quadriceps did not differ between RIPC and CON conditions during fatiguing isometric knee extension exercise. |
| Telles et al. (2022) | M16, recreationally trained volunteers | 1 RM testing on bench press, front latissimus pull-down, shoulder press, leg press 45º, hack machine, and smith squat | IPC | 4 x 5 min at 220 mmHg (alternate arms) | + | No significant differences were found in the comparison between IPC, SHAM, and CON for HR, LF_nu_, HF_nu_, LF/HF ratio, and RMSSD_ms_.. |
| Telles et al. (2021) | M16, recreationally trained volunteers | Bench press, leg press, lat pulldown, hack machine squat, shoulder press, smith squat at 80% 1RM to concentric failure (Strength Training) | IPC | 4 x 5 min at 220 mmHg (alternate arms) | NA | A significant decrease in LF_nu_ and LF/HF ratio was observed from 60 min post for IPC vs SHAM and IPC vs CON. A significant increase in HF_nu_ was observed from 60 min post for IPC vs SHAM and IPC vs CON. A significant increase in RMSSD_ms_ was found from post 60 for IPC vs SHAM. |
| ter Beek et al. (2020) | M15, healthy volunteers | Incremental exercise test (Cycling) | IPC | 4 x 5 min at 250 mmHg (thighs) | ~ | During incremental exercise testing, IPC had no effect on exercising HR, TSI, or HHb compared to SHAM. |
| Tocco et al. (2015) | M11, trained runners | 5 km TT (Running) | IPC | 3 x 5 min at 50 mmHg > SBP (thighs) | ~ | There was no effect of IPC on exercising HR or oxygen pulse during 5 km running compared to the SHAM and REF. |
| Tomschi et al. (2018) | M10, healthy volunteers | Incremental exercise test (Cycling) | RIPC | 4 x 5 min at 200 mmHg (right arm) | ~ | There was no effect of IPC, PLA, or SHAM on RBC deformability during incremental exercise testing. |
| Turnes et al. (2018) | M16, regional- and national-level rowers | 2000 m TT (Rowing) | IPC | 3 x 5 min at 220 mmHg (alternate thighs)  3 x 10 min at 220 mmHg (alternate thighs) | ~ | During exercise, IPC10 significantly reduced ΔTSI and ΔO2Hb concentration compared with the CON. IPC5 resulted in lower ΔO2Hb at the 2000m segment compared to IPC10. No significant differences were found between treatments for △HHb during the 2000m test. Both IPC10 and IPC5 increased HR over the first 500 m of the TT. |
| Valenzuela et al. (2019) | M16, resistance trained volunteers | Force-velocity/rep-to-failure at 60% 1 RM (Bench Press) | IPC | 3 x 5 min at 220 mmHg (unilateral arm) | ~ | A significant decrease in the skin temperature of the pectoralis and biceps muscles was observed after the intervention and before the warmup in IPC but not SHAM. During exercise, there were similar increases in Tsk in the pectoralis muscle in both conditions. |
| Wiggins et al. (2019) | M13, endurance athletes | 5 km TT in normoxia and hypoxia + Constant Load Test (Cycling) | IPC | 4 x 5 min at 220 mmHg (alternate thigh) | + | IPC increased ΔHHb compared to SHAM during moderate-intensity constant load exercise in hypoxia. During the 5 km TTs, there were no differences in ΔHHb, ΔO_2_Hb, ΔTSI, or $\dot{\text{Q}}$_CAP_ between trials in normoxia and hypoxia. |
| Zinner et al. (2017) | M7; F6, moderately-to-well-trained team sport athletes | 16 x 30 m multidirectional sprint (Running) | IPC + RIPC | 3 x 5 min at 240 mmHg (thighs)  3 x 5 min at 180 - 190 mmHg (arms) | ~ | There were no differences in HR, nor re- and de-oxygenation of SmO_2 of_ the vastus lateralis and biceps brachii during repeat sprints between IPC, RIPC, or CON conditions. |
| Abbreviations: ASR = active strain rate ;BP = blood pressure; BP_max_ = maximum blood pressure; BP_rest_ = resting blood pressure; CK = creatine kinase; CON = control; DAP = diastolic arterial pressure; DBP = diastolic blood pressure; E’ = peak early diastolic myocardial velocity; E/é ratio = early diastolic filling to early diastolic myocardial velocity ratio; EDV = end diastolic volume; EF = ejection fraction; EIPC = ischemic preconditioning applied after exercise; FMD = flow mediated dilation; GET = gas exchange threshold; HF_nu_ = high frequency domain in normalized units; HHb = deoxygenated haemoglobin; ΔHHb = change in deoxygenated haemoglobin; HHb TD + τ = time delay from the nadir of the initial decline in the deoxygenated haemoglobin response; HR = heart rate; HR_mean_ = mean heart rate; HR_max_ = maximum heart rate; HR_peak_ = peak heart rate; HRR = heart rate recovery; HRR30 = heart rate recovery at 30 s post-exercise; HRR60 = heart rate recovery at 60 s post-exercise ; HRV = heart rate variability; hs-troponin = high sensitive troponin I; IPC = ischemic preconditioning; IPC+nitrate = ischemic precondition with nitrate supplementation; IPC+PEMI = ischemic preconditioning with post-exercise muscle ischemia; IPC+placebo = ischemic preconditioning with a placebo supplement; IPC+RE = ischemic precondition and resistance exercise; IPC & RIPC = both local and remote ischemic preconditioning applied simultaneously; IPC + RIPC = local and remote ischemic preconditioning applied on separate visits; IPC5 = ischemic preconditioning @ 3 x 5 min intervals; IPC10 = ischemic preconditioning @ 3 x 10 min intervals; IPC45 = ischemic preconditioning applied 45 min before exercise; KE = knee extension; LF_nu_ = low frequency domain in normalized units; LF/HF ratio = low-frequency high-frequency ratio; LnHfa = absolute high-frequency power; LnSDNN = standard deviation of normal R-R intervals; LOP = limb occlusion pressure; LV = left ventricle = LVEDV = left ventricular end diastolic volume; LVET = left ventricular ejection time; MAP = mean arterial pressure; MAP_max_ = maximum mean arterial pressure; MVC = maximum voluntary contraction; m$\dot{\text{V}}$O_2_ = muscle oxygen consumption; NIRS = near infrared spectroscopy; NT-proBNP = N terminal pro B-type natriuretic peptide; O_2_Hb = oxygenated haemoglobin; ΔO_2_Hb = change in oxygenated haemoglobin; peak ε = peak regional strain; PEMI = post-exercise muscle ischemia; PLA = placebo; PPO = peak power output; $\dot{\text{Q}}$ = cardiac output; $\dot{\text{Q}}$_max_ = maximum cardiac output; $\dot{\text{Q}}$_peak_ = peak cardiac output; $\dot{\text{Q}}$_CAP_ = microvascular capillary blood flow; RIPC = remote ischemic preconditioning; RBC = red blood cell; RE = resistance exercise; REF = reference test; RMSSDms = square root of the sum of the square of the differences between the R-R intervals divided by the number of R-R intervals ;RVFAC = right ventricle fractional area change; SaO_2_ = arterial oxygen saturation; ΔSaO_2_ = change in arterial oxygen saturation; SAP = systolic arterial pressure; SBP = systolic blood pressure; SHAM+RE = sham treatment with resistance exercise; SmO_2_ = muscle oxygen; SpO_2_ = peripheral oxygen saturation; SSR = systolic strain rate ; SV = stroke volume; SV/LVET = stroke volume and left ventricular ejection time ratio; SV_max_ = maximum stroke volume; SV_peak_ = peak stroke volume; SVR = systemic vascular resistance; SVR_max_ = maximum systemic vascular resistance; tHb = total haemoglobin; ΔtHb = change in total haemoglobin; TSI = tissue saturation index; ΔTSI = change in tissue saturation index; Tsk = skin temperature; TT = time trial; TTE = time to exhaustion; $\dot{\text{V}}$O_2_ = oxygen consumption; T30 = time constant of heart rate decay $\dot{\text{V}}$O_2max_ = maximum oxygen consumption; $\dot{\text{V}}$O_2peak_ = peak oxygen consumption; *W*max = maximum workload; W_peak_ = peak workload; 1 RM = one repetition maximum; 6MWT = six minute walk test; 24-IPC = ischemic preconditioning 24 h before test; + = positive effect of IPC on exercise performance outcomes; ~ = no change in exercise performance outcomes after IPC; - = negative effect of IPC on exercise performance outcomes. | | | | | | |

| **Supplementary Table 2 - Metabolic Responses to Acute Local or Remote Ischemic Preconditioning** | | | | | | |
| --- | --- | --- | --- | --- | --- | --- |
| **Study** | **Subjects** | **Exercise** | **Type of IPC** | **Protocol** | **Effect on performance (+/-/~)** | **Findings** |
| Andreas et al. (2011) | [M14; M9](applewebdata://A3A0583F-4D05-4F29-A64E-9E8FA282573C/../Thesis/Andreas%20-%20Effect%20of%20ischemic%20preconditioning%20in%20skeletal%20muscle%20measured%20by%20functional%20megnetic%20resonance%20imaging%20and%20spectroscopy.pdf), healthy volunteers | [Isometric plantarflexion MVC](applewebdata://A3A0583F-4D05-4F29-A64E-9E8FA282573C/../Thesis/Andreas%20-%20Effect%20of%20ischemic%20preconditioning%20in%20skeletal%20muscle%20measured%20by%20functional%20megnetic%20resonance%20imaging%20and%20spectroscopy.pdf) | [IPC](applewebdata://A3A0583F-4D05-4F29-A64E-9E8FA282573C/../Thesis/Andreas%20-%20Effect%20of%20ischemic%20preconditioning%20in%20skeletal%20muscle%20measured%20by%20functional%20megnetic%20resonance%20imaging%20and%20spectroscopy.pdf) | 3 x 5 min at 200 mmHg (right thigh) | NA | During the ischemic phase of IPC, the PCr signal decreased robustly and recovered rapidly during the reperfusion period. During post-ischemic stenosis, PCr increased only slightly. The BOLD signal intensity decreased during ischemia, ischemic exercise, and post-ischemic stenosis, but increased during hyperemic reperfusion. When IPC was applied 4 h prior to ischemia, maximal PCr reperfusion signals significantly increased and mitigated the peak BOLD signal during reperfusion. |
| Baikoğlu and Kaldirimci, (2019) | M14, healthy volunteers | Wingate Anaerobic Test (Cycling) | IPC | 1 x 5 min at undisclosed pressure (thighs) | ~ | There was no effect of IPC on lactic acid threshold levels before ischemia, immediately after ischemia, or 3 minutes after ischemia in the dominant leg. There was no effect of IPC on lactic acid levels after the Wingate anaerobic power test. |
| Bailey et al. (2012b) | M13, healthy volunteers | Incremental exercise test + 5 km TT (Running) | IPC | 4 x 5 min at 220 mmHg | + | IPC and SHAM led to similar submaximal gas parameters during running exercise. The increase in submaximal blood lactate concentrations was lower in IPC compared to SHAM. There was no effect of IPC on $\dot{\text{V}}$O_2_, $\dot{\text{V}}$E, or RER at varying incremental running speeds. |
| Caru et al. (2019a) | M8; F7, amateur triathletes | Steady state test at 75% and 115% GET (Cycling) | RIPC | 4 x 5 min at 50 mmHg > SBP (right arm) | ~ | Compared to CON, there was no effect of RIPC on the gain of the primary and slow component, time constants of the primary and slow component, time delay between the primary and slow component, and amplitude of the slow component of $\dot{\text{V}}$O_2_ kinetics during steady-state exercise. |
| Carvalho and Barroso, (2019) | M10, resistance-trained volunteers | Dynamic knee extension to failure at 85% 1RM | IPC | 4 x 5 min at 250 mmHg (alternate thighs) | + | There was no difference between IPC and SHAM on Δblood lactate following a single set of KE exercise to failure at 85% 1RM |
| Ceylan and Franchini, (2022) | M10, elite judo athletes | Special judo fitness test | IPC | 3 x 5 min at 220 mmHg (alternate thighs) | ~ | After the judo-specific test, blood lactate concentrations were lower in the IPC condition compared to the CON condition. |
| Chen et al. (2022) | M16, 400 m runners | TTE at 110% $\dot{\text{V}}$O_2max_ (Running) | IPC + RIPC | 4 x 5 min at 220 mmHg (alternating arms/thighs) | + | During supramaximal treadmill running at 110% $\dot{\text{V}}$O_2max_, both IPC and RIPC conditions increased the MAOD compared with CON. There were no differences in MAOD between local IPC and RIPC maneuvers. Blood lactate concentrations were similar between all three conditions. |
| Cheng et al. (2021) | M15, college basketball players | Repeat Wingate Anaerobic Test (Cycling) | IPC + RIPC | 4 x 5 min at 220 mmHg (thighs) | + | There were no differences between local IPC, RIPC, SHAM, and CON for baseline or post-exercise blood lactate and pH concentrations or exercising $\dot{\text{V}}$O_2peak_ during sprint interval exercise. |
| Cheung et al. (2020) | M8; F8, recreationally active volunteers | Incremental exercise test (Cycling) | IPC | 4 x 5 min at LOP (alternate thigh) | + | Baseline, and submaximal, and peak exercise $\dot{\text{V}}$O_2_ and blood lactate concentration values were not different between IPC, SHAM, and CON conditions. |
| Clevidence et al. (2012) | M12, amateur cyclists | Incremental exercise test (Cycling) | IPC | 3 x 5 min at 220 mmHg (alternate thighs) | ~ | During cycling exercise at increasing submaximal intensities, no differences were found in peak or average $\dot{\text{V}}$O_2_, $\dot{\text{V}}$E, or RER between IPC and CON conditions. Moreover, pre-exercise, exercising, and post-exercise blood glucose and blood lactate concentrations were not different between IPC and CON. |
| Cocking et al. (2018b) | 12 trained cyclists (gender not specified) | 375 kJ TT (Cycling) | IPC + RIPC | 4 x 5 min at 220 mmHg (thighs) 8 x 5 min at 220 mmHg (thighs)  4 x 5 min at 220 mmHg (thigh)  4 x 5 min at 220 mmHg (arms) | + | During cycling TTs, traditional IPC was associated with likely trivial higher blood lactate responses and possibly beneficial $\dot{\text{V}}$O2 responses compared to SHAM. None of the other IPC maneuvers demonstrated differences in metabolic responses at various time points in the TT. |
| Crisafulli et al. (2011) | M17, healthy volunteers | Incremental exercise test + TTE (Cycling) | IPC | 3 x 5 min at 50 mmHg > SBP (thighs) before or after exercise | + | There were no differences in resting $\dot{\text{V}}$O_2_, $\dot{\text{V}}$CO_2_, VE, or A-VO_2_D between IPC, EIPC, and REF test. $\dot{\text{V}}$O_2max_ and VCO_2max_ were not different between any of the three treatments during incremental exercise testing. VE_max_ was significantly higher after IPC and EIPC. During tests of anaerobic capacity $\dot{\text{V}}$O_2max_, $\dot{\text{V}}$CO_2max_, $\dot{\text{V}}$E_max_ A-VO_2_D_max_, and peak blood lactate values were not different between REF, IPC, and EIPC. |
| Cruz et al. (2015) | 12 recreationally trained cyclists (gender not specified) | TTE at 100% PPO (Cycling) | IPC | 4 x 5 min at 220 mmHg (thighs) | + | There was no effect of IPC on baseline $\dot{\text{V}}$O_2_ or blood lactate concentrations. During maximal constant load cycling, IPC increased $\dot{\text{V}}$O_2peak_ due to a higher amplitude of the slow component of $\dot{\text{V}}$O_2_ kinetics. Accumulated VO_2_ was also increased by IPC relative to CON. There were no effects of IPC on other variables of $\dot{\text{V}}$O_2_ kinetics or $\dot{\text{V}}$E. End exercise lactate concentrations were significantly higher after IPC compared to the CON condition. Changes in $\dot{\text{V}}$O_2peak_ and the slow component of $\dot{\text{V}}$O_2_ kinetics largely correlated with the observed performance improvements via IPC. |
| Cruz et al. (2016) | M15, recreationally trained cyclists | 60 s Sprint (Cycling) | IPC | 4 x 5 min at 220 mmHg (thighs) | + | IPC had no effect on pre-test $\dot{\text{V}}$O_2_, $\dot{\text{V}}$O_2peak_, or accumulated VO_2_ during a 60 s cycle sprint compared to CON. IPC increased the AOD and EPOC compared to CON. There were no differences in blood lactate concentrations at rest, immediately post-sprint, or peak lactate concentrations between IPC and CON. Blood lactate immediately pre-exercise was significantly increased in IPC. The absolute amplitude of blood lactate kinetics was also elevated by IPC relative to the CON |
| da Mota et al. (2019) | M13, healthy volunteers | 2 x 5 km TT (Cycling) | IPC | 3 x 5 min at 220 mmHg (thighs | + | IPC did not influence pre or post TT blood lactate concentrations or SpO2 % compared to a SHAM during successive 5 km cycling TTs. |
| de Groot et al. (2010) | M12; F3, well-trained cyclists | Incremental exercise test (Cycling) | IPC | 3 x 5 min at 220 mmHg (thighs) | + | During incremental exercise tests, IPC significantly increased $\dot{\text{V}}$O_2max_ relative to the CON condition. There was no effect of IPC on submaximal $\dot{\text{V}}$O_2_ at 50 W, 100 W, and 150 W. There was no effect of IPC on RQ, $\dot{\text{V}}$E, or peak blood lactate concentrations compared to CON. |
| Ferreira et al. (2016) | M8; F7, university swimmers | 6 x 50 m sprint (Swimming) | IPC | 3 x 5 min at 220 mmHg (thighs) | + | No differences were found in blood lactate concentrations between IPC and SHAM following repeated swimming sprint efforts. |
| Franz et al. (2018) | M19, healthy volunteers | 3 x 10 biceps curls at 80% 1RM | IPC | 3 x 5 min at 200 mmHg (arms) | NA | IPC blunted the increase in eccentric exercise-induced CK levels 24, 48, and 72 h post-exercise compared to the CON condition. |
| Gibson et al. (2015) | M7; F9, invasion sport athletes | 5 x 6 s sprint (Cycling) | IPC | 3 x 5 min at 220 mmHg (alternate thighs) | ~ | During repeat sprint cycling exercise, there was no effect of IPC on peak blood lactate concentrations compared to PLA and CON. In female participants only, blood lactate was reduced after IPC compared to PLA and CON. |
| Griffin et al. (2019) | M12, recreationally active volunteers | 3 min sprint (Cycling) | IPC | 4 x 5 min at 220 mmHg (thighs) | + | During an all-out 3 min cycle sprint, IPC had no effect on $\dot{\text{V}}$O_2peak_, $\dot{\text{V}}$O_2_ MRT, and peak blood lactate concentrations compared to a SHAM. |
| Griffin et al. (2018) | M12, recreationally active volunteers | Repeat sprint (Running) | IPC + RIPC | 4 x 5 min at 220 mmHg (arms + thighs) | ~ | During repeated sprint running, neither IPC nor RIPC had an effect on blood lactate concentrations compared to SHAM. |
| Halley et al. (2020) | M8, well trained kayakers | Repeated 1000m simulated kayak races (Kayaking) | IPC | 4 x 5 min at 220 mmHg (alternate thighs) | + | During successive 1000m kayak ergometer TTs, there was no observed effect of IPC1 or IPC2 on blood lactate, pH, or SaO2 compared to CON. There was no effect of IPC1 or IPC2 on AOD or mean $\dot{\text{V}}$O_2_ during the TTs. |
| James et al. (2016) | M11, recreational club runners | Incremental exercise test at 32°C (Running) | IPC | 4 x 5 min at 220 mmHg (thighs) | ~ | IPC had no effect on submaximal running speeds at 2 and 4 mMol L blood lactate and did not affect blood glucose concentrations during incremental exercise tests in the heat. IPC did not influence $\dot{\text{V}}$O_2max_, blood lactate, or muscle temperature during exercise. IPC did increase v$\dot{\text{V}}$O_2max_, and reduced TCORE during exercise. |
| Jean-St-Michel et al. (2011) | M8; F8, national and internationally competitive swimmers | 7 x 200 m submaximal + 100/200 m TT (Swimming) | RIPC | 4 x 5 min at 15 mmHg > SBP (arms) | + | During submaximal and maximal swimming trials, IPC did not increase lactate production compared to CON. |
| Kaur et al. (2017) | M12; F6, recreationally active runners | Incremental submaximal (Running) | IPC | 3 x 5 min at 220 mmHg (thighs) | ~ | At submaximal running intensities, IPC did not influence $\dot{\text{V}}$O_2_, $\dot{\text{V}}$E, RER, EE, or blood lactate concentrations compared to SHAM. |
| Kido et al. (2015) | M15, recreationally active volunteers | Work-to-work test (Cycling) | IPC | 3 x 5 min at > 300 mmHg (thighs) | + | During the work-to-work test, IPC had no effect on baseline, end-exercise, primary amplitude, primary dime delay, primary time constant, or mean response time $\dot{\text{V}}$O_2_ values at moderate-intensity and severe-intensity exercise compared to CON. There was no effect of IPC on 240 sec, slow phase time delay, slow phase amplitude, and end exercise $\dot{\text{V}}$O_2_ kinetics during severe-intensity exercise. IPC significantly lowered $\dot{\text{V}}$O_2_ amplitude and $\dot{\text{V}}$O_2_ MRT at moderate-intensity exercise. Baseline $\dot{\text{V}}$O2 was lower after IPC in severe intensity exercise. There was no effect of IPC on resting, pre-exercise, end of low-intensity exercise, end of moderate-intensity exercise, exhaustion, or Δ blood lactate values between IPC and CON conditions. |
| Kilding et al. (2018) | M9, well-trained cyclists | Incremental exercise test + 4 km TT (Cycling) | IPC | 4 x 5 min at 200 mmHg (thighs) | ~ | IPC did not influence $\dot{\text{V}}$O_2peak_, GET, O_2_ cost of exercise, or exercise economy during incremental exercise testing. There was no effect of IPC on baseline, end-exercise, phase II time constant, mean response time, primary amplitude, or primary gain $\dot{\text{V}}$O_2_ kinetics at moderate intensity (80% GET) compared to SHAM. IPC reduced end-exercise $\dot{\text{V}}$O_2_, slow phase amplitude, slow component amplitude, and overall gain $\dot{\text{V}}$O_2_ kinetics during heavy intensity exercise compared to SHAM. |
| Kraus et al. (2015) | M27; F16, recreationally active volunteers | 4 x 30 s Wingate Anaerobic Test (Cycling) | RIPC | 4 x 5 min at undisclosed pressure (left arm study 1; bilateral arm study 2) | + | During repeat Wingate tests, neither unilateral nor bilateral RIPC influenced blood lactate concentrations compared to the SHAM condition. |
| Lisbôa et al. (2017) | M11, Competitive swimmers | 3 x 50 m TT (Swimming) | IPC & RIPC | 4 x 5 min at 220 mmHg (thighs)  4 x 5 min at 180 mmHg (arms) | + | IPC increased blood lactate accumulation after 50 m swimming TTs completed 2 h and 8 h after application compared to CON. There was no effect of IPC on blood lactate accumulation 1 h after IPC administration. |
| Lopes et al. (2018) | M15, recreationally active volunteers | Repeated shuttle sprint (Running) | IPC | 3 x 5 min at 220 mmHg (alternate thighs) | ~ | During repeated shuttle sprint running, IPC had no effect $\dot{\text{V}}$O_2peak_, $\dot{\text{V}}$CO_2peak_, RER, peak or Δ blood lactate concentrations compared to CON. |
| Marocolo et al. (2016b) | M13, recreationally trained volunteers | 12 RM leg extension | IPC | 4 x 5 min at 220 mmHg (alternate thighs) | + | Following KE exercise to failure, there were no differences in blood lactate concentrations between IPC, SHAM, and CON conditions. |
| Marocolo et al. (2016a) | M21, recreationally trained volunteers | 12 RM elbow flexion | IPC + RIPC | 4 x 5 min at 220 mmHg (altenate arms/alternate thighs) | + | Following elbow flexion exercise to failure there were no differences in blood lactate concentrations between IPC, SHAM, and CON conditions. |
| Marocolo et al. (2017) | M13, amateur soccer players | Incremental shuttle run (Running) | IPC | 4 x 5 min at 220 mmHg (alternate thighs) | ~ | There were no differences between IPC, SHAM, or CON conditions in blood lactate concentrations after completing the YoYo Intermittent Endurance Test. |
| Martin et al. (2021) | M14; F1, chronic heart failure patients | Six minute walk test (walking) | RIPC | 4 x 5 min at 20 mmHg > SBP (right arm) | ~ | During the 6MWT, there were no differences in $\dot{\text{V}}$O_2_, $\dot{\text{V}}$CO2, $\dot{\text{V}}$E, or RER between IPC and SHAM conditions in patients with chronic heart failure. |
| McIlvenna et al. (2019) | M10, competitive cyclists | Incremental exercise test + 16.1 km TT (Cycling) | IPC | 4 x 5 min at 180 mmHg (thighs) | ~ | During submaximal exercise, neither IPC+Nitrate nor IPC+PLA supplementation influenced $\dot{\text{V}}$O_2rest_, $\dot{\text{V}}$O_2exercise_, $\dot{\text{V}}$O_2_ MRT, $\dot{\text{V}}$O_2_ amplitude, or functional gain compared to baseline. |
| Mota et al. (2020) | F20, healthy volunteers | 3 min sprint (Arm Cycling) | IPC | 3 x 3 min (2 min off) at 50 mmHg > SBP (arms) | ~ | IPC decreased pre-exercise blood lactate concentrations compared to CON. IPC had no effect on post-exercise blood lactate or Δblood lactate concentrations in response to 3 min all-out arm cycling exercise. |
| Paixão et al. (2014) | 15 competitive cyclists (gender not specified) | 3 x Wingate Anaerobic Test (Cycling) | IPC | 4 x 5 min at 250 mmHg (alternate thighs) | - | During repeat Wingate tests, IPC was detrimental to total anerobic power compared to CON. There were no differences found between IPC and CON for post-exercise blood lactate concentrations. |
| Paradis-Deschênes et al. (2020) | M9, trained cyclists, runners, and triathletes | Repeat 5 km TT (Cycling) | IPC | 3 x 5 min at 220 mmHg (alternate thighs) between 5 km TTs | + | IPC applied in recovery between 5 km TTs showed no difference in bicarbonate concentrations, base excess of blood and total concentration of CO_2_, or SpO_2_ when compared to other recovery techniques such as active recovery and neuromuscular electrical stimulation. |
| Paradis-Deschênes et al. (2017) | M9; F8, strength trained volunteers | 5 x 5 MVC knee extension | IPC | 3 x 5 min at 200 mmhg (right thigh) | + | Contraction metabolic efficiency ratio was not meaningfully different between IPC and SHAM conditions or between sexes during maximal force resistance exercise. |
| Patterson et al. (2015) | M14, recreationally active volunteers | 12 x 6 s repeat sprinting (Cycling) | IPC | 4 x 5 min at 220 mmHg (thighs) | + | IPC had no effect on resting or exercise $\dot{\text{V}}$O_2_ values during repeated sprint cycling efforts. Blood lactate values were possibly higher at sprint 4, sprint 8, and sprint 12 in the IPC condition when compared to the PLA condition. |
| Paull and van Guilder, (2019) | M6; F4, NCAA Division I Track middle-distance runners | Supramaximal TTE (Running) | RIPC | 4 x 5 min at 220 mmHg (right arm) | + | RIPC increased AOD during supramaximal running TTE compared to SHAM and BASELINE conditions. Resting blood lactate concentrations were significantly decreased by RIPC compared to SHAM. |
| Sabino-Carvalho et al. (2017) | M14; F4, competitive  middle- long-distance runners | Discontinuous incremental exercise (Running) | IPC | 4 x 5 min at 220 mmHg (alternate thighs) | ~ | During discontinuous incremental running exercise IPC did not influence $\dot{\text{V}}$O_2_, $\dot{\text{V}}$CO_2_, $\dot{\text{V}}$E, blood lactate, LT, and the O_2_ cost of running compared to SHAM and CON conditions. |
| Seeger et al. (2017) | M10; F2, healthy volunteers | 5 km TT (Running) | IPC | 4 x 5 min at 220 mmHg (thighs) before or 24 h before exercise | ~ | Post 5 km running TT blood lactate concentrations were not different between IPC, 24IPC, and SHAM conditions. |
| Slysz and Burr, (2018) | M12, healthy volunteers | Wingate Anaerobic Test + incremental exercise test (Cycling) | IPC | 3 x 5 min at 220 mmHg (thighs) | + | IPC had no effect on submaximal $\dot{\text{V}}$O_2_ or $\dot{\text{V}}$O_2max_ during incremental exercise test compared to baseline, IPC+walk, and IPC+EMS. |
| ter Beek et al. (2020) | M15, healthy volunteers | Incremental exercise test (Cycling) | IPC | 4 x 5 min at 250 mmHg (thighs) | ~ | IPC had no effect on submaximal $\dot{\text{V}}$O_2_ or $\dot{\text{V}}$O_2max_ during incremental exercise test compared to SHAM. Post-exhaustion blood lactate concentrations were not different between IPC and SHAM conditions. |
| Tocco et al. (2015) | M11, runners | 5 km TT (Running) | IPC | 3 x 5 min at 50 mmHg > SBP (thighs) | ~ | During 5 km running TTs, there was no effect of IPC on the aerobic energy cost of running, $\dot{\text{V}}$O_2_, $\dot{\text{V}}$CO_2_, RER, $\dot{\text{V}}$E, oxygen pulse, or blood lactate concentrations compared to the REF test or SHAM conditions. |
| Tomschi et al. (2018) | M10, healthy volunteers | Incremental exercise test (Cycling) | RIPC | 4 x 5 min at 200 mmHg (right arm) | ~ | There was no effect of IPC on watt power at 2 and 4 mMol/L LT compared to SHAM or PLA conditions. Peak blood lactate concentrations were not different between SHAM, IPC, and PLA for pooled, responders, or non-responders. |
| Turnes et al. (2018) | M16, regional- and national-level rowers | 2000 m TT (Rowing) | IPC | 3 x 5 min at 220 mmHg (alternate thighs)  3 x 10 min at 220 mmHg (alternate thighs) | ~ | In response to 2000 m rowing TTs, there were no alterations observed in $\dot{\text{V}}$O_2_, $\dot{\text{V}}$O_2peak_, or blood lactate concentrations between IPC5, IPC10, and CON conditions. |
| Wiggins et al. (2019) | M13, endurance athletes | 5 km TT in normoxia and hypoxia (Cycling) | IPC | 4 x 5 min at 220 mmHg (alternate thigh) | + | In response to 5 km cycling TTs in normoxia and hypoxia, there was no effect of IPC on $\dot{\text{V}}$O_2_, or SpO2%, between IPC and SHAM conditions. |
| Williams et al. (2018) | M14; F6, national- and international swimmers | 100m TT (Swimming) | IPC | 4 x 5 min at LOP (thighs) | ~ | After 100 m swimming TTs, base excess, pH, bicarbonate, total CO_2_, sO_2_% and blood lactate concentrations were not influence by IPC applied 2 hr or 24 hrs before exercise compared to CON. |
| Zinner et al. (2017) | M7; F6, moderately-to-well-trained teamsport athletes | 16 x 30 m multidirectional sprint (Running) | IPC + RIPC | 3 x 5 min at 240 mmHg (thighs)  3 x 5 min at 180 - 190 mmHg (arms) | ~ | During multidirectional sprint running, there was no difference in $\dot{\text{V}}$O_2_, RER, or $\dot{\text{V}}$E between RIPC, IPC, and CON conditions. |
| Abbreviations: AOD = accumulated oxygen deficit; A-VO_2_D = artero-venous oxygen difference; A-VO_2_D_max_ = maximum artero-venous oxygen difference; BOLD = blood oxygen level-dependent signal; CK = creatine kinase; CON = control; CO_2_ = carbon dioxide; EE = energy expenditure; EIPC = ischemic preconditioning applied after exercise; EPOC = excess post-exercise oxygen consumption; GET = gas exchange threshold; IPC = ischemic preconditioning; IPC1 = IPC applied before time trial 1; IPC2 = IPC applied before time trial 1 and between time trial 1 and 2; IPC5 = ischemic preconditioning @ 3 x 5 min intervals; IPC10 = ischemic preconditioning @ 3 x 10 min intervals; IPC+EMS = ischemic preconditioning combined with electric muscle stimulation; IPC+Nitrate = ischemic preconditioning with nitrate supplementation; IPC+Placebo = ischemic preconditioning with placebo supplementation; IPC & RIPC = both local and remote ischemic preconditioning applied simultaneously; IPC + RIPC = local and remote ischemic preconditioning applied on separate visits; IPC+walk = ischemic preconditioning combined with walking; KE = knee extension; LOP = limb occlusion pressure; LT = lactate threshold; MAOD = maximum accumulated oxygen deficit; MVC = maximal voluntary contraction; O_2_ = oxygen; PCr = phosphocreatine; PLA = placebo; PPO = peak power output; REF = reference test; RER = respiratory exchange ratio; RIPC = remote ischemic preconditioning; RQ = respiratory quotient; SaO_2_ = arterial oxygen saturation; sO_2_ = capillary oxygen saturation; SpO_2_ = peripheral oxygen saturation; TCORE = core temperature; TT = time trial; TTE = time to exhaustion; $\dot{\text{V}}$CO_2_ = carbon dioxide production; $\dot{\text{V}}$CO_2max_ = maximum carbon dioxide production; $\dot{\text{V}}$CO_2peak_ = peak carbon dioxide production; $\dot{\text{V}}$O_2_ = oxygen consumption; $\dot{\text{V}}$O_2exercise_ = exercising oxygen consumption; $\dot{\text{V}}$O_2max_ = maximum oxygen consumption; $\dot{\text{V}}$O_2peak_= peak oxygen consumption; $\dot{\text{V}}$O_2rest_ = resting oxygen consumption; $\dot{\text{V}}$O_2_ MRT = oxygen consumption mean response time; v$\dot{\text{V}}$O_2max_ = velocity at maximum oxygen consumption; $\dot{\text{V}}$E = ventilation; $\dot{\text{V}}$E_max_ = maximum ventilation; 1 RM = one repetition maximum; 6MWT = six minute walk test; 12 RM = twelve repetition maximum; 24IPC = ischemic preconditioning applied 24 h before test; | | | | | | |

| **Supplementary Table 3 – Nervous System Responses to Acute Local or Remote IPC** | | | | | | |
| --- | --- | --- | --- | --- | --- | --- |
| **Study** | **Subjects** | **Exercise** | **Type of IPC** | **Protocol** | **Effect on performance (+/-/~)** | **Findings** |
| Angius et al. (2022) | M13; F4, healthy volunteers | 3 min KE at 70% *W*max + PEMI | IPC | 3 x 5 min at 220 mmHg (exercising thigh) | NA | IPC reduced MAP during metaboreflex activation. Reduced MAP was likely the consequence of reduced EDV. No differences were found to PPT, whereas MP was significantly lower during PEMI in IPC compared to SHAM. |
| Behrens et al. (2020) | M16, recreationally active volunteers | Isometric KE TTE at 20% MVC | IPC | 3 x 5 min at LOP (thighs) | ~ | During fatiguing isometric KE exercise, performance fatiguability, maximal voluntary torque, voluntary activation, and muscle activity were not different between IPC and SHAM conditions. In responders only, performance improvements were associated with larger impairments in neuromuscular function. That is a larger change in maximum voluntary torque and voluntary activation, compared to the SHAM condition. |
| Carvalho and Barroso, (2019) | M10, Resistance-trained volunteers | Isometric knee extension MVC test | IPC | 4 x 5 min at 250 mmHg (alternate thighs) | + | During isometric KE MVC testing, there were no differences in EMG mean power frequency or EMG muscle activation between IPC and SHAM conditions. |
| Cocking et al. (2021) | M11, trained cyclists | 10 x 6 s repeated sprint w/ 24 s recovery (Cycling) | IPC | 4 x 5 min at 220 mmHg (thighs) | ~ | During repeat sprint cycling performance, IPC had no effect on EMG RMS at the vastus lateralis, biceps femoris, or rectus femoris muscles compared to the SHAM condition. |
| Cruz et al. (2015) | 12 recreationally trained cyclists (gender not specified) | TTE at 100% PPO (Cycling) | IPC | 4 x 5 min at 220 mmHg (thighs) | + | During maximal constant load cycling, IPC significantly increased progressive EMG activity of the vastus lateralis compared to the CON condition |
| Cruz et al. (2016) | M15, recreationally trained cyclists | 60 s Sprint (Cycling) | IPC | 4 x 5 min at 220 mmHg (thighs) | + | During maximal cycling sprint exercise, IPC significantly increased EMG amplitude of the vastus lateralis compared to the CON condition. EMG amplitude was higher at the 15 – 20 s and 40 – 55 s time intervals. At the 40 – 55 s time interval, there was a significant increase in the EMG/PO ratio in IPC compared to CON. |
| Halley et al. (2018) | M11, resistance trained volunteers | Isometric knee extension 2 min MVC | IPC | 3 x 5 min at 220 mmHg (alternate thighs) | ~ | During fatiguing isometric KE exercise, there were no differences in MVC, voluntary activation, sEMG amplitude or RERave for VL or VM between IPC and CON conditions. No differences were observed between IPC and CON with respect to maximal torque, maximal rate of torque production, or resting twitch characteristics. |
| Halley et al. (2019) | M11, resistance trained volunteers | 6 x 11 MVC knee extension in normoxia and hypoxia | IPC | 3 x 5 min a 220 mmHg (alternate thighs) | ~ | During KE exercise in normoxia and hypoxia, MVC, twitch torque, and voluntary activation were not different between IPC and SHAM conditions. |
| Horiuchi et al. (2015) | M15, healthy volunteers | Dynamic handgrip exercise at 10% and 25% MVC | IPC | 4 x 5 min at 220 mmHg (unilateral arm) | NA | IPC augmented the decrease in O_2_Hb in response to a cold pressor test. During hand grip exercise at 25% MVC, O_2_Hb in response to cold pressor test increased significantly more when preceded by IPC than CON. IPC did not affect HR, MAP, or skin blood flow at rest or during exercise. |
| Hyngstrom et al. (2018) | M4; F6, chronic stroke survivors | Knee extensor isometric MVC | IPC | 5 x 5 min at 225 mmHg (unliateral thigh) | + | Acute IPC increased paretic leg strength whereas no difference was observed in the SHAM condition. Increased strength was accompanied by increased magnitude of muscle EMG during MVC and a decrease in motor unit recruitment thresholds during submaximal contractions. |
| Incognito et al. (2017) | M13, healthy volunteers | 2 min static hand grip exercise at 30% MVC + 3 min PECO | IPC | 3 x 5 min at 200 mmHg (left arm) | NA | Resting MAP was reduced in the IPC condition following PECO compared to the CON condition, while resting MSNA burst frequency was unchanged by either condition. MAP, HR, SV, Q, and TVC during SHG and PECO were the same PRE and POST following IPC and SHAM conditions. MSNA burst frequency, burst incidence, and total MSNA responses during SHG and PECO were not different PRE and POST between IPC and SHAM conditions. |
| Marshall et al. (2020) | M18,  recreationally active volunteers | 5 x 5 repeat sprints at 150% max workload (Cycling | IPC | 3 x 5 min at 220 mmHg (alternate thighs) | ~ | During repeated sprint cycling exercise, IPC did not have an effect on peak twitch amplitude, time-to-peak twitch, maximal M-wave, voluntary activation, EMG amplitude, maximal rate of rise in EMG, V-wave, or H-reflexes compared to the SHAM condition. |
| Morley et al. (2021) | M8; F5, recreationally active volunteers | 4 x 5 min constant load cycling + orthostatic challenge | IPC | 4 x 5 min at LOP (thighs) | NA | The normal RIPC condition reduced resting HR and supressed HR 30 s following orthostatic challenge in comparison to the CON condition. RIPC_aug_ elevated HR compared to both CON and RIPC prior to and during the orthostatic challenge. LnSDNN and LnHfa power were reduced by RIPCaug on the morning of the treatment compared to RIPC and CON. |
| Mulliri et al. (2016) | M14, healthy volunteers | Dynamic handgrip exercise at 45% MVC TTE | IPC | 3 x 5 min at 50 mmHg > SBP (unilateral arm) | NA | After IPC, the MAP response was reduced compared with the PEMI test. Reduced MAP was due to reduced SV and VFR during the IPC-PEMI test compared to the PEMI test without IPC. |
| Patterson et al. (2015) | M14, recreationally active volunteers | 12 x 6 s repeat sprinting (Cycling) | IPC | 4 x 5 min at 220 mmHg (thighs) | + | During repeat sprint cycling performance, IPC possibly increased the rate of change in MDF and EMG compared to PLA. |
| Pereira et al. (2020) | M10; F10, healthy volunteers | Isometric plantarflexion TTE at 20% MVC | IPC & RIPC | 3 x 5 min at 225 mmHg (non-dominant thigh/upper arm) | + | In responses to isometric plantarflexion TTE, males demonstrated greater MAP compared to females after receiving IPC compared to SHAM. Voluntary activation and presynaptic inhibition of leg Ia afferents were not altered after IPC for males or females compared to SHAM. |
| Pethick et al. (2021) | M6; F4, healthy volunteers | Intermittent isometric knee extension contractions to failure at 40% MVC | IPC | 3 x 5 min at 224 mmHg (unilateral thigh) | + | During fatiguing KE exercise the rate of decrease in complexity was significantly lower after IPC compared to SHAM. The rate of increase in EMG amplitude was significantly slower after IPC compared to SHAM. |
| Sabino-Carvalho et al. (2019) | M11; F4, competitive middle- long-distance runners | Discontinuous incremental exercise (Running) | IPC | 4 x 5 min at 220 mmHg (alternate thighs) | NA | During incremental running exercise, respiratory sinus arrhythmia and HRV were not different between IPC, SHAM, and CON conditions at pre- and post-intervention assessments. T30 was similar among interventions. IPC increased HRR30 s at 70% and 75% of maximal effort versus SHAM and CON conditions. IPC did not change resting cardiac vagal control but did boost fast post-exercise cardiac vagal reactivation at exercise intensities below the lactate threshold. |
| Tanaka et al. (2016) | M12, healthy volunteers | Isometric knee extension TTE at 20% MVC | IPC | 3 x 5 min at > 300 mmHg (right thigh) | + | During fatiguing isometric KE exercise, quadriceps EMG activity was not different between IPC and CON conditions. |
| Tanaka et al. (2020) | M14, healthy volunteers | Isometric knee extension TTE at 20% MVC | RIPC | 3 x 5 min at 300 mmHg (left/non-exercising thigh) | ~ | During fatiguing isometric KE exercise, quadriceps EMG activity was not different between RIPC and CON conditions. |
| Telles et al. (2022) | M16, recreationally trained volunteers | 1 RM testing on bench press, front latissimus pull-down, shoulder press, leg press 45º, hack machine, and smith squat | IPC | 4 x 5 min at 220 mmHg (alternate arms) | + | During 1 RM testing, no differences were found between IPC, SHAM, and CON conditions for LFnu, HFnu, LF/HF ratio, and RMSSDms. |
| Telles et al. (2021) | M16, recreationally trained volunteers | Bench press, leg press, lat pulldown, hack machine squat, shoulder press, smith squat at 80% 1 RM to concentric failure (Strength Training) | IPC | 4 x 5 min at 220 mmHg (alternate arms) | NA | Following fatiguing resistance exercise, LFnu and LF/HF ratio were significantly decreased 60 min post-exercise following IPC compared to SHAM and CON conditions. HFnu was significantly increased 60 min post-exercise in IPC vs SHAM and CON, respectively. Similarly, 60 min post-exercise there was a significant increase in RMSSDms following IPC compared to the SHAM condition. |
| Abbreviations: CON = control condition; EMG = electromyography; EMG/PO ratio = electromyography to power output ratio; HF_nu_ = high frequency domain in normalized units; HR = heart rate; HRR30s = heart rate recovery at 30 s post-exercise; HRV = heart rate variability; IPC = ischemic preconditioning; IPC-PEMI = ischemic preconditioning plus post exercise muscle ischemia; IPC & RIPC = both local and remote ischemic preconditioning applied simultaneously; KE = knee extensor; LF_nu_ = low frequency domain in normalized units; LF/HF ratio = low frequency to high frequency ratio; LnHfa = absolute high frequency power; LnSDNN = standard deviation of normal R-R intervals; LOP = limb occlusion pressure; MAP = mean arterial pressure; MDF = median frequency; MP = muscle pain; MSNA = muscle sympathetic nerve activity; MVC = maximum voluntary contraction; O_2_Hb = oxygenated haemoglobin; PECO = post exercise circulatory occlusion; PEMI = post exercise muscle ischemia; PPO = peak power output; PPT = pain pressure threshold; Q = cardiac output; RERave = maximal rate of surface electromyography rise; RIPC = remote ischemic preconditioning; RIPCaug = remote ischemic preconditioning applied while cycling; RMS = root mean squared; RMSSDms = square root of the sum of the square of the differences between the R-R intervals divided by the number of R-R intervals; sEMG = surface electromyography; SHG = static hand grip; SV = stroke volume; TTE = time to exhaustion; TVC = total vascular conductance; T30 = time constant of heart rate decay; VFR = ventricular filling rate; VL = vastus lateralis; VM = vastus medialis; *W*max = maximum workload; 1 RM = one repetition maximum; | | | | | | |

| **Supplementary Table 4 - Perceptual Responses to Acute Local or Remote IPC** | | | | | | |
| --- | --- | --- | --- | --- | --- | --- |
| **Study** | **Subjects** | **Exercise** | **Type of IPC** | **Protocol** | **Effect on performance (+/-/~)** | **Findings** |
| Angius et al. (2022) | M13; F4, healthy volunteers | 3 min KE at 70% *W*max + PEMI | IPC | 3 x 5 min at 220 mmHg (exercising thigh) | NA | During fatiguing KE exercise, there was no difference in MP, or RPE between IPC and SHAM conditions. During PEMI, MP was significantly lower in the IPC condition compared to the SHAM. |
| Bailey et al. (2012a) | M13, healthy volunteers | Graded exercise test + 5 km TT (Running) | IPC | 4 x 5 min at 220 mmHg (thighs) | + | During the graded exercise test and the 5 km TTs, IPC had no influence on post-exercise RPE. |
| Bailey et al. (2012b) | M13, healthy volunteers | Graded exercise test + 5 km TT (Running) | IPC | 4 x 5 min at 220 mmHg (thighs) | + | During the graded exercise test IPC had no influence on RPE. During the 5 km TT, RPE was significantly lower in IPC compared to CON after the first 1 km. There were no differences in RPE between IPC and CON at 2 km, 3 km, 4 km, or 5 km. |
| Behrens et al. (2020) | M16, recreationally active volunteers | Isometric knee extension TTE at 20% MVC | IPC | 3 x 5 min at LOP (thighs) | ~ | Compared to SHAM, IPC lowered RPE during fatiguing isometric KE exercise in responders only. |
| Cocking et al. (2017) | 14 recreationally to well-trained cyclists (gender not specified) | 1 h TT (Cycling) | IPC & RIPC | 4 x 5 min at 220 mmHg (alternate thighs/arms) | ~ | IPC did not affect RPE during 1 h cycling TTs. |
| Cocking et al. (2018b) | 12 trained cyclists (gender not specified) | 375 kJ TT (Cycling) | IPC + RIPC | 4 x 5 min at 220 mmHg (thighs) 8 x 5 min at 220 mmHg (thighs)  4 x 5 min at 220 mmHg (thigh)  4 x 5 min at 220 mmHg (arms) | + | During cycling TTs, none of the IPC maneuvers affected RPE at any of the recorded time points of the TTs compared to a SHAM condition. |
| Cruz et al. (2015) | 12 recreationally trained cyclists (gender not specified) | TTE at 100% PPO (Cycling) | IPC | 4 x 5 min at 220 mmHg (thighs) | + | During cycling TTE at 100% $\dot{\text{V}}$O2max, IPC attenuated the rate of increase in RPE compared to CON. |
| da Mota et al. (2019) | M13, healthy volunteers | 2 x 5 km TT (Cycling) | IPC | 3 x 5 min at 220 mmHg (thighs | + | IPC did not affect RPE during successive 5 km cycling TTs compared to SHAM. |
| da Silva Novaes et al. (2020) | M16, recreationally active volunteers | Whole body RE training to failure at 80% 1RM | IPC | 4 x 5 min at 220 mmHg (alternate arms) | + | During resistance exercise repetitions to failure, IPC did not affect RPE compared to CON and SHAM conditions. |
| de Souza et al. (2021) | M9, moderately trained volunteers | KE exercise to failure at 75% 1RM + Maximum voluntary isometric contraction test | IPC | 3 x 5 min at 50 mmHg > SBP (right thigh) | ~ | IPC did not affect RPE or perceived recovery during KE exercise. |
| Ferreira et al. (2016) | M8; F7, university swimmers | 6 x 50 m sprint (Swimming) | IPC | 3 x 5 min at 220 mmHg (thighs) | + | During repeat swimming sprints, IPC did not affect RPE compared to SHAM. |
| Franz et al. (2018) | M19, healthy volunteers | 3 x 10 biceps curls at 80% 1RM | IPC | 3 x 5 min at 200 mmHg (arms) | NA | In response to eccentric exercise-induced muscle damage, IPC significantly reduced pain ratings compared to CON. |
| Garcia et al. (2017) | M8, subelite rugby players | Agility T-Test + countermovement jump + 30 s jump test | IPC | 3 x 5 min at 220 mmHg (thighs) | ~ | RPE was not different between IPC and CON conditions during rugby specific exercise protocol. |
| Gibson et al. (2015) | M7; F9, invasion sport athletes | 5 x 6 s sprint (Cycling) | IPC | 3 x 5 min at 220 mmHg (alternate thighs) | ~ | During repeat sprint cycling, IPC did not affect RPE compared to PLA or CON conditions. |
| Griffin et al. (2019) | M12, recreationally active volunteers | Repeat sprint (Running) | IPC + RIPC | 4 x 5 min at 220 mmHg (arms + thighs) | ~ | During repeat sprint running, IPC did not affect RPE when compared to SHAM. |
| Halley et al. (2018) | M11, resistance trained volunteers | Isometric knee extension 2 min MVC | IPC | 3 x 5 min at 220 mmHg (alternate thighs) | ~ | During fatiguing isometric exercise, IPC did not affect total work, MVC, twitch torque, voluntary activation, or pain scores using the visual analog scale compared to SHAM or CON. Peak HHb and tHb concentrations were elevated in IPC relative to CON. |
| Halley et al. (2020) | M8, well trained kayakers | Repeated 1000m simulated kayak races (Kayaking) | IPC | 4 x 5 min at 220 mmHg (alternate thighs) | + | During successive 1000 m kayak ergometer TTs, IPC did not have an effect on RPE compared to CON. |
| Hittinger et al. (2014) | M15, highly trained cyclists | Incremental exercise test at sea level and simulated altitude (Cycling) | IPC | 4 x 5 min at 10 - 20 mmHg > SBP (thighs) | ~ | During incremental exercise tests at sea level and simulated altitude, IPC did not have an effect on RPE compared to SHAM conditions. |
| Kaur et al. (2017) | M12; F6, recreationally active runners | Incremental submaximal (Running) | IPC | 3 x 5 min at 220 mmHg (thighs) | ~ | During incremental submaximal running, IPC did not affect RPE compared with the SHAM condition. |
| Lalonde and Curnier, (2015) | M8; F9, recreationally active volunteers | 6 s Sprint + Wingate Anaerobic Test (Cycling) | RIPC | 4 x 5 min at 50 mmHg > SBP (right arm) | ~ | IPC had no influence on RPE during the Wingate anaerobic test. |
| Marocolo et al. (2016a) | M21, recreationally trained volunteers | 12 RM elbow flexion | IPC + RIPC | 4 x 5 min at 220 mmHg (altenate arms/alternate thighs) | + | During fatiguing elbow flexion exercise, IPC did not affect RPE compared to SHAM or REF conditions. |
| Marocolo et al. (2017) | M13, amateur soccer players | Incremental shuttle run (Running) | IPC | 4 x 5 min at 220 mmHg (alternate thighs) | ~ | During incremental shuttle running, IPC did not affect RPE compared to SHAM and CON conditions. |
| Mota et al. (2020) | F20, healthy volunteers | 3 min sprint (Arm Cycling) | IPC | 3 x 3 min (2 min off) at 50 mmHg > SBP (arms) | ~ | In response to a 3 min all-out arm cycling sprint, RPE was not different between IPC and SHAM conditions. |
| Paradis-Deschênes et al. (2018) | M13, trained cyclists | 5 km TT at low + moderate + high altitude (Cycling) | IPC | 3 x 5 min at 220 mmHg (alternate thighs) | + | During 5 km cycling TTs as low altitude, IPC administration had no effect on RPE. When IPC was administered before 5 km cycling TTs at moderate altitude, RPE was significantly lower compared to the SHAM condition. |
| Patterson et al. (2015) | M14, recreationally active volunteers | 12 x 6 s repeat sprinting (Cycling) | IPC | 4 x 5 min at 220 mmHg (thighs) | + | IPC did not influence RPE at any time points during repeated sprint cycling compared to the PLA condition. |
| Pereira et al. (2020) | M10; F10, healthy volunteers | Isometric plantarflexion TTE at 20% MVC | IPC & RIPC | 3 x 5 min at 225 mmHg (thigh and arm) | + | During isometric plantarflexion TTE exercise, IPC increased time to task failure in male participants only, and this performance improvement was correlated with lower response to pressure pain. During exercise, there were no differences in RPE between IPC, SHAM, and CON conditions. |
| Sabino-Carvalho et al. (2017) | M14; F4, competitive middle- long-distance runners | Discontinuous incremental exercise (Running) | IPC | 4 x 5 min at 220 mmHg (alternate thighs) | ~ | IPC did not influence RPE during discontinuous running exercise compared to CON or SHAM conditions. |
| Seeger et al. (2017) | M10; F2, healthy volunteers | 5 km TT (Running) | IPC | 4 x 5 min at 220 mmHg (thighs) before or 24 h before exercise | ~ | Neither IPC nor 24IPC affected RPE during 5 km running TTs compared to the SHAM condition. |
| Slysz and Burr, (2021) | M8; F5, aerobically trained volunteers | 5 km TT (Cycling) | IPC | 3 x 5 min at LOP (thighs) | ~ | Compared to the CON condition, IPC did not acutely affect pain intensity during a cold-water immersion test but it did reduce the total time spent under pain. |
| Telles et al. (2020) | M16, resistance trained volunteers | 3 x 80% 1 RM bench press/leg press TTE | IPC | 4 x 5 min at 220 mmHg (alternate thigh) | + | During fatiguing resistance exercise, IPC did not have an effect on RPE compared to the SHAM condition. |
| ter Beek et al. (2020) | M15, healthy volunteers | Incremental exercise test (Cycling) | IPC | 4 x 5 min at 250 mmHg (thighs) | ~ | Compared to the SHAM condition, IPC attenuated RPE during cycling at 210 W and 245 W. |
| Turnes et al. (2018) | M16, regional- and national-level rowers | 2000 m TT (Rowing) | IPC | 3 x 5 min at 220 mmHg (alternate thighs)  3 x 10 min at 220 mmHg (alternate thighs) | ~ | There was no effect of IPC5 or IPC10 on RPE during 2000m rowing TTs compared to the CON condition. |
| Abbreviations: CON = control trial; IPC = ischemic preconditioning; IPC & RIPC = both local and remote ischemic preconditioning applied simultaneously; IPC + RIPC = local and remote ischemic preconditioning applied on separate visits; IPC5 = ischemic precondition @ 3 x 5 min intervals; IPC10 = ischemic preconditioning at 3 x 10 min intervals; KE = knee extensor; LOP = limb occlusion pressure; MP = muscle pain; MVC = maximum voluntary contraction; PEMI = post exercise muscle ischemia; PPO = peak power output; RE = resistance exercise; REF = reference test; RIPC = remote ischemic preconditioning; RPE = rating of perceived exertion; TT = time trial; TTE = time to exhaustion; $\dot{\text{V}}$O_2max_ = maximum oxygen consumption; *W*max = maximum workload; 1 RM = one repetition maximum; 12 RM = twelve repetition maximum; 24IPC = ischemic preconditioning applied 24 h before testing. | | | | | | |
